# An assessment of public health surveillance of Zika virus infection and potentially associated outcomes in Latin America

**DOI:** 10.1186/s12889-018-5566-7

**Published:** 2018-05-24

**Authors:** Leonelo E. Bautista, Víctor M. Herrera

**Affiliations:** 1 Department of Population Health Sciences, School of Medicine and Public Health, University of Wisconsin at Madison, 610 Walnut Street, WARF 703, Madison, WI 53726-2397 USA; 2 Center for Biomedical Research, Universidad Autónoma de Bucaramanga, Bucaramanga, Colombia

## Abstract

**Background:**

We evaluated whether outbreaks of Zika virus (ZIKV) infection, newborn microcephaly, and Guillain-Barré syndrome (GBS) in Latin America may be detected through current surveillance systems, and how cases detected through surveillance may increase health care burden.

**Methods:**

We estimated the sensitivity and specificity of surveillance case definitions using published data. We assumed a 10% ZIKV infection risk during a non-outbreak period and hypothetical increases in risk during an outbreak period. We used sensitivity and specificity estimates to correct for non-differential misclassification, and calculated a misclassification-corrected relative risk comparing both periods. To identify the smallest hypothetical increase in risk resulting in a detectable outbreak we compared the misclassification-corrected relative risk to the relative risk corresponding to the upper limit of the endemic channel (mean + 2 SD). We also estimated the proportion of false positive cases detected during the outbreak. We followed the same approach for microcephaly and GBS, but assumed the risk of ZIKV infection doubled during the outbreak, and ZIKV infection increased the risk of both diseases.

**Results:**

ZIKV infection outbreaks were not detectable through non-serological surveillance. Outbreaks were detectable through serologic surveillance if infection risk increased by at least 10%, but more than 50% of all cases were false positive. Outbreaks of severe microcephaly were detected if ZIKV infection increased prevalence of this condition by at least 24.0 times. When ZIKV infection did not increase the prevalence of severe microcephaly, 34.7 to 82.5% of all cases were false positive, depending on diagnostic accuracy. GBS outbreaks were detected if ZIKV infection increased the GBS risk by at least seven times. For optimal GBS diagnosis accuracy, the proportion of false positive cases ranged from 29 to 54% and from 45 to 56% depending on the incidence of GBS mimics.

**Conclusions:**

Current surveillance systems have a low probability of detecting outbreaks of ZIKV infection, severe microcephaly, and GBS, and could result in significant increases in health care burden, due to the detection of large numbers of false positive cases. In view of these limitations, Latin American countries should consider alternative options for surveillance.

**Electronic supplementary material:**

The online version of this article (10.1186/s12889-018-5566-7) contains supplementary material, which is available to authorized users.

## Background

Disease surveillance is an essential tool for the development of effective public health and patient care policies. During the last two years, Latin American countries have implemented surveillance of Zika virus (ZIKV) infection, newborn microcephaly, and Guillain-Barré syndrome (GBS) in response to ongoing outbreaks and concerns about possible causal associations between these diseases. In most countries, these systems follow guidelines from international health agencies [1, 2], and are commonly based on the passive detection and report of cases by health care personnel to surveillance units in Ministries of Health. Surveillance reports suggest substantial increases in microcephaly prevalence and GBS incidence following ZIKV outbreaks [3, 4], but increases could be explained by intensified surveillance [5].

ZIKV-microcephaly-GBS surveillance systems have been in place for a short time and there are no data on their performance. We present an assessment of the expected performance of these systems, based on current knowledge and assumptions about disease frequency, accuracy of diagnostic tests, and hypothetical effects of ZIKV infection on microcephaly and GBS risk. Specifically, we evaluated under what conditions would outbreaks of these diseases be identifiable, and what would be the impact of false positive cases detected through surveillance on health care burden. Findings from this study could inform the implementation of surveillance systems in Latin America.

## Methods

### Overall approach

We evaluated the expected performance of a hypothetical surveillance system in a population of 10 million with a birth rate of 17.3/1000 population. These figures are close to the median population size and fertility rate in Latin American countries. We conducted separate evaluations for ZIKV infection, newborn microcephaly, and GBS surveillance.

**Fig. 1:**
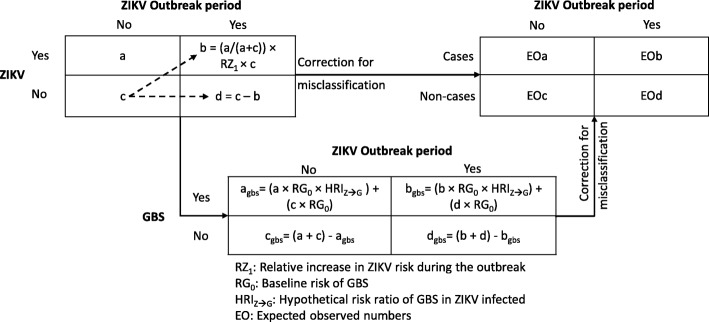
Approach for comparing risk of Zika virus infection and related outcomes during outbreak and non-outbreak period

We assumed the risk of ZIKV infection during a baseline, non-outbreak period (RZ_0_), was 10%, and individuals who got infected were no longer susceptible in a subsequent outbreak period [6]. We calculated the ZIKV infection risk during an outbreak (RZ_1_) as RZ_0_ × RRZ_0 → 1_, where RRZ_0 → 1_ is a hypothetical relative increase in risk, and generated “true” 2 × 2 tables of period by ZIKV infection (Fig. 1 and Additional file 1, item 1). Then, we obtained 2 × 2 tables corrected for misclassification of ZIKV infection status and calculated the expected observed risk ratio (EORR) and the expected observed case ratio (EOCR) comparing the outbreak and non-outbreak period. By progressively increasing it from one to nine, we identified the minimum RRZ_0 → 1_ resulting in a detectable outbreak. We also calculated the proportion of false positive cases (PFP) identified through surveillance, an indicator of the added health care burden resulting from errors in ZIKV infection diagnosis.

We used the same approach to assess a possible GBS/microcephaly outbreak during a ZIKV infection outbreak with RZ_1_ = 2 × RZ_0_. However, we accounted for the number of new cases of ZIKV infection in our calculation of the risk of GBS/microcephaly during the outbreak. For instance, for GBS the risk during the outbreak period (RG_1_) was calculated as [(RG_0_ × NZ_(−)_) + (RG_0_ × NZ_(+)_ × HRI_Z → G_)] / (NZ_(−)_ + NZ_(+)_)], where RG_0_ is the baseline risk of GBS, HRI_Z → G_ is the relative increase in GBS risk among ZIKV infected individuals, and NZ_(+)_ and NZ_(−)_ are the numbers of ZIKV infected and non-infected individuals during the outbreak, respectively. We obtained misclassification corrected 2 × 2 tables and calculated the EORR, EOCR, and PFP for each condition. To find a minimum resulting in a detectable outbreak, we probed for values of HRI_Z → G_ of 1, 5, and 10 for GBS and for values of HRI_Z → M_ of 1, 5, 10 and 15 for microcephaly.

Outcome misclassification-corrected 2 × 2 tables were obtained using standard formulae for the case of non-differential errors [7]. Specifically, if A = number of exposed cases, B = number of non-exposed cases, C = number of exposed non-cases, and D = number of non-exposed non-cases from the “true” 2 × 2 table, then the expected observed number of exposed cases would be [(A × test sensitivity) + (C × (1 - test specificity))]. Also, the expected observed number of non-exposed non-cases would be [(D × test specificity) + (B × (1 - test sensitivity))]. We assumed no changes in case definition or surveillance procedures occurred during the outbreak.

Surveillance guidelines do not specify when to declare an outbreak [1, 8, 9]. We applied the standard criterion of an incidence beyond two standard deviations above the baseline average, the upper limit of the endemic channel, as the cut point to identify an outbreak [10]. In consequence, we declared an outbreak happened if the *p*-value from a chi square test for the EORR was below 0.0227.

#### Zika virus infection

The surveillance case definition used in Latin America: “*Patient with rash with at least two or more of the following signs or symptoms: fever, usually <38.5 °C, conjunctivitis, arthralgia, myalgia, and peri-articular edema*” [1], resembles the definition used by Duffy et al. in a study of an outbreak on Yap Island, Micronesia [11]. Duffy et al. reviewed medical records in all health centers to identify suspected cases and used ELISA tests for IgM antibodies and RT-PCR for ZIKV and dengue virus to confirm the diagnosis. They also conducted a serological survey in a random sample of the population. We used their data and formulae for screening studies with extreme verification bias [12, 13] to estimate the sensitivity and the specificity of the surveillance case definition in three scenarios (Additional file 1, item 2).

In the regular surveillance scenario only cases requesting medical care were detected, similar to Duffy et al.’s study [11], and sensitivity and specificity were 2 and 96%, respectively. The enhanced surveillance scenario was similar to regular surveillance, but we assumed the case detection probability was five times higher in Latin America than in Yap Island [11]. In this case, sensitivity was 9.8% and specificity was 79.7%. In the serological surveillance scenario all suspected cases of ZIKV infection were detected through a survey and infection was confirmed using the same tests used in the Yap Island study [11]. In this case, sensitivity was 37.7% and specificity was 81.1%.

#### Microcephaly

We conducted separate assessments for all and severe microcephaly, traditionally defined as a head circumference (HC) < 2 and < 3 standard deviations (SD) below the mean, respectively [14, 15]. Under these definitions the prevalences of all and severe microcephaly are 22.75 and 1.35/1000 newborn, respectively. We assumed ZIKV infection increased the risk of microcephaly in the baseline and outbreak period but only in newborn of women infected in that period.

We simulated HC values to estimate accuracy of a diagnosis of microcephaly (Additional file 1, item 3). First, we generated “true” HC values by randomly drawing one million observations from the HC distribution in Brazilian newborn (mean 34.2 cm, SD 1.2) [16]. We obtained random errors from a normal distribution with mean 0 and SD equal to the intra-observer technical error of HC measurements (TEM) [17] in the WHO Multicentre Growth Reference Study [18], and added them to the true values [19]. We cross-classified individuals by applying the definition of all and severe microcephaly to the true (gold standard) and the error augmented values, and calculated the sensitivity and specificity of HC measurements. We also used estimates of sensitivity and specificity for all microcephaly, among low birth weight newborn (≤2000 g) from Bhushan et al. [20], and their TEM values to estimate sensitivity and specificity for severe microcephaly (see Table 2 for values of sensitivity and specificity).

#### Guillain-Barré syndrome

We used a GBS incidence of 2/100,000 in our calculations. This value was taken from a published review in which incidence ranged from 0.4 to 4 cases/100,000 population in all but two out of 34 studies [21].

GBS diagnostic certainty could be very low, particularly in the early stages of the disease, and there is no clinical characteristic or biomarker that perfectly discriminates GBS from mimicking neurologic disorders [22]. The Brighton criteria is the standard tool to classify GBS cases by diagnostic certainty [23–25]. A certainty level ≤ 3, recommended for surveillance case definition, was used in this analysis [1]. We estimated an average sensitivity of 82.1% from three published studies [23, 24, 26] (Additional file 1, item 4) and used specificities of 91.7, 88.9, and 80.6%, from the sole study on this issue, as far as we know [27]. However, we applied this specificity only to individuals with incident peripheral neuropathy, the key clinical feature that most commonly leads to a suspicion of GBS [22, 28, 29], since only they could be falsely diagnosed as GBS cases. We used a random effects model to estimate the average incidence of peripheral neuropathy in published studies [30–40] and used the low and upper limits of its 95% confidence interval in the analysis (3.3 and 5.6/10,000; Additional file 1, item 5).

## Results

### Zika virus

A minimum RRZ_0 → 1_ resulting in a detectable outbreak was not identifiable for the scenarios of regular and enhanced surveillance. In both cases, the EORR and the EOCR were always less than one and decreased progressively with increasing RRZ_0 → 1_ (Table 1; Additional file 1, item 6). For instance, for enhanced surveillance the EORR and EOCR were 0.95 and 0.85 for RRZ_0 → 1_ = 2 and 0.78 and 0.70 for RRZ_0 → 1_ = 5, respectively. As expected, the PFP decreased with higher RRZ_0 → 1_. However, under the scenario of enhanced surveillance the PFP reached 92.3% for RRZ_0 → 1_ = 2 and 83.5% for RRZ_0 → 1_ = 5. Thus, for these scenarios, only 1 out of 13 and 1 out of 6 cases detected through enhanced surveillance were true ZIKV infection cases.

**Table 1:** Expected Observed Risk Ratios and Case Ratios and Proportion of False Positive Cases of Zika Virus Infection During an Outbreak, by Type of Surveillance, Increase in Infection Risk, and Case Definition Sensitivity and Specificity

	RRZ_0→1_^a^	Case definition sensitivity	Case definition specificity	Expected observed risk ratio	Expected observed case ratio	False positive proportion
Regular surveillance^b^	1	2.0	95.9	1.00	0.90	94.89
	2	2.0	95.9	0.95	0.85	92.27
	5	2.0	95.9	0.78	0.70	83.52
	8	2.0	95.9	0.62	0.56	73.11
Enhanced surveillance^c^	1	9.8	79.7	1.00	0.90	94.89
	2	9.8	79.7	0.95	0.85	92.27
	5	9.8	79.7	0.78	0.70	83.52
	8	9.8	79.7	0.62	0.56	73.11
Serologic surveillance^d^	1	37.7	81.1	1.00	0.90	81.86
	2	37.7	81.1	1.09	0.98	74.36
	5	37.7	81.1	1.36	1.23	55.17
	8	37.7	81.1	1.63	1.47	39.77

^a^Hypothetical Relative Increase in Infection Risk (RRZ_0 → 1_); ^b^Surveillance case definition and demand of health care similar to those observed in Yap Island [11]; ^c^Surveillance case definition similar to the one used in Yap Island with a five-fold increase in the demand of health care (detection probability); ^d^ Surveillance based of serologic surveys of random samples of the population

In the scenario of serologic based surveillance, an outbreak was detectable for RRZ_0 → 1_ ≥ 1.10. In that case, the EORR was 1.01, but the EOCR was 0.91. Moreover, the EOCR was > 1 only for RRZ_0 → 1_ ≥ 2.4. Even for larger values of the RRZ_0 → 1_ the EORR and the EOCR were strongly biased towards the null. For instance, for RRZ_0 → 1_ = 5 the EORR and the EOCR were 1.36 and 1.23, and the PFP was 55%. Thus, even when the risk of ZIKV increased 5-fold during the outbreak, less than half of all cases of ZIKV infection detected through serologic surveillance were true positive cases.

### Microcephaly

Outbreaks of all microcephaly were detectable for HRI_Z → M_ ≥ 2.00, regardless of the level of sensitivity and specificity of the diagnosis, but the expected observed prevalence ratio (EOPR) was less than 1.10 in all cases (Table 2; Additional file 1, item 7). In contrast, outbreaks of severe microcephaly were detectable only for HRI_Z → M_ ≥ 24.00, but the EOPR was only 1.48. Even when the HRI_Z → M_ was as high as 15, the EOPR was ≤1.41 for both all and severe microcephaly, regardless of sensitivity and specificity (Tables 3 and 4; Additional file 1, item 8). When ZIKV infection did not increase microcephaly prevalence (HRI_Z → M_ = 1), the PFP for all microcephaly and severe microcephaly ranged from 22.6 to 56.6% and from 34.7 to 82.5%, respectively, depending on the sensitivity and specificity of HC measurements (Tables 3 and 4).

**Table 2:** Minimum Hypothetical and Expected Observed Prevalence Ratio to Detect an Outbreak of Microcephaly if Zika Virus Infection Risk Doubles, by Sensitivity and Specificity of Microcephaly Case Detection

Microcephaly	TEM ^a^(cm)	Sensitivity (%)	Specificity (%)	Prevalence ratio	
				Hypothetical	Expected Observed
All	0.24	85.1	99.4	2.00	1.06
	0.42	77.6	98.8	2.00	1.04
	0.71	91.2	97.2	2.00	1.03
Severe	0.24	81.9	99.9	24.00	1.48
	0.42	75.0	99.9	27.50	1.43
	0.71	65.9	99.6	39.50	1.33

^a^ TEM: Technical error of head circumference measurements. TEM = 0.71 came from Bhushan et al. (J Clin Epidemiol Vol. 44 (10):1027–1035, 1991), but values of sensitivity and specificity for all microcephaly were taken directly from their article, while those for severe were obtained by simulation. All other TEM values came from Onis (Acta Pædiatrica, 2006; Suppl 450:38–46) and were used to estimate sensitivity and specificity by simulation

**Table 3:** Expected Observed Prevalence Ratio of All Microcephaly and Proportion of False Positive Cases During an Outbreak of Zika Virus Infection, by Increase in the Prevalence of Microcephaly in Newborn of Infected Mothers, and Sensitivity and Specificity of Microcephaly Case Detection

HRI_Z → M_ in prevalence ratio^a^	Intra-observer TEM^b^	Prevalence ratio ^c^	Sensitivity (%)	Specificity (%)	Expected observed prevalence ratio	Proportion of false positives (%)
1	0.24	1.00	85.1	99.4	1.00	22.6
5	0.24	1.23	85.1	99.4	1.19	15.6
10	0.24	1.38	85.1	99.4	1.33	11.1
15	0.24	1.47	85.1	99.4	1.41	8.6
1	0.71	1.00	91.8	97.2	1.00	56.6
5	0.71	1.23	91.8	97.2	1.12	45.2
10	0.71	1.38	91.8	97.2	1.22	35.9
15	0.71	1.47	91.8	97.2	1.30	29.6

^a^ HRI_Z → M_: Hypothetical relative increase in the prevalence of microcephaly

^b^ TEM: Intraobserver technical error of measurement of head circumference

^c^ Prevalence ratio of microcephaly (outbreak vs non-outbreak period) under perfect diagnostic sensitivity and specificity

**Table 4:** Expected Observed Prevalence Ratio of Severe Microcephaly and Proportion of False Positive Cases During an Outbreak of Zika Virus Infection, by Increase in the Prevalence of Microcephaly in Newborn of Infected Mothers, and Sensitivity and Specificity of Microcephaly Case Detection

HRI_Z → M_ in prevalence ratio^a^	Intra-observer TEM^b^	Prevalence ratio ^c^	Sensitivity (%)	Specificity (%)	Expected observed prevalence ratio	Proportion of false positives (%)
1	0.24	1.00	81.9	99.9	1.00	34.7
5	0.24	1.21	81.9	99.9	1.14	24.7
10	0.24	1.35	81.9	99.9	1.26	18.4
15	0.24	1.50	81.9	99.9	1.41	14.7
1	0.71	1.00	65.9	99.6	1.00	82.5
5	0.71	1.21	65.9	99.6	1.06	74.5
10	0.71	1.35	65.9	99.6	1.10	66.8
15	0.71	1.50	65.9	99.6	1.16	60.5

^a^ HRI_Z → M_: Hypothetical relative increase in the prevalence of microcephaly

^b^ TEM: Intraobserver technical error of measurement of head circumference

^c^ Prevalence ratio of microcephaly (outbreak vs non-outbreak period) under perfect diagnostic sensitivity and specificity

### Guillain-Barré syndrome

Under ideal conditions, when the sensitivity and specificity for detecting GBS cases were both 100%, outbreaks of GBS were detected when HRI_Z → G_ ≥ 4.0 (Table 5; Additional file 1, item 9). However, the minimum HRI_Z → G_ resulting in a detectable outbreak increased for decreasing values of specificity. For the estimated sensitivity of 82.1%, outbreaks were detected if HRI_Z → G_ ≥ 7, HRI_Z → G_ ≥ 8, and HRI_Z → G_ ≥ 9 for specificities of 91.7, 88.9, and 80.6%, respectively.

**Table 5:** Minimum Hypothetical Relative Increase in Risk (HRI_Z → G_) to Detect an Outbreak of Guillain-Barré Syndrome and Expected Observed Risk Ratios if the Risk if Zika Virus Infection Doubles During an Outbreak^a^

Sensitivity/specificity (%) ^b^	HRI_Z → G_ in GBS risk^c^	Observed risk ratio
100/100	4	1.18
82.1/100	5	1.23
82.1/91.7	7	1.30
82.1/88.9	8	1.33
82.1/80.6	9	1.36

^a^ Corrected for non-differential misclassification due to a sensitivity of 82.1% and varying levels of specificity of the GBS case definition

^b^ A fixed level of sensitivity was obtained by averaging findings from studies assessing the validity of Brighton criteria in adults

^c^ HRI_Z → G_: Minimum Hypothetical Relative Increase in Risk of Guillain-Barré síndrome

When ZIKV infection was not associated with GBS risk (HRI_Z → G_ = 1), the PFP varied with specificity, from 34 to 59% and from 50 to 72%, for low and high incidence of peripheral neuropathy, respectively (Table 6; Additional file 1, item 10). Regardless of HRI_Z → G_ value, the misclassification-corrected EORR were small and decreased with decreasing specificity. Indeed, for specificity of 100% and HRI_Z → G_ = 10, the EORR was only 1.47. For the lowest specificity the minimum PFP was 33.8% or higher in all scenarios. For higher values of specificity, the PFP was below 30%, regardless of the HRR, but only if the incidence of peripheral neuropathy was low. When the incidence of peripheral neuropathy was high, the PFP ranged from 24.5 to 61.0%, if HRI_Z → G_ > 1.

**Table 6:** True and Expected Observed Risk Ratio of Guillain-Barré Syndrome (GBS) During an Outbreak of Zika Virus (ZIKV) Infection, by Hypothetical Increase in GBS Risk Among ZIKV Infected Individuals and by Specificity and Sensitivity of the GBS Case Definition Used for Surveillance

Hypothetical risk ratio	Specificity (%)	Incidence peripheral neuropathy	True GBS risk ratio ^a^	Expected observed risk ratio ^b^	PFP ^c^ (%)	Number of false positive GBS cases
1	100.0	3.3/10,000	1.00	1.00	0.0	0
1	91.7				54.5	454
1	88.9				62.4	631
1	80.6				75.2	1153
5	100.0	3.3/10,000	1.29	1.29	0.0	0
5	91.7			1.18	40.7	414
5	88.9			1.16	49.4	591
5	80.6			1.12	64.8	1113
10	100.0	3.3/10,000	1.47	1.47	0.0	0
10	91.7			1.33	29.2	364
10	88.9			1.30	37.9	540
10	80.6			1.23	54.6	1063
1	100.0	5.6/10,000	1.00	1.00	0.0	0
1	91.7				68.4	822
1	88.9				74.7	1122
1	80.6				84.1	2012
5	100.0	5.6/10,000	1.29	1.29	0.0	0
5	91.7			1.14	56.4	782
5	88.9			1.12	64.2	1082
5	80.6			1.08	76.5	1971
10	100.0	5.6/10,000	1.47	1.47	0.0	0
10	91.7			1.27	45.3	731
10	88.9			1.24	53.8	1032
10	80.6			1.17	68.5	1921

^a^ Risk ratio of GBS during the ZIKV outbreak, assuming sensitivity of 82.1% and specificity of 100% for the diagnosis of GBS; ^b^ Risk ratio of GBS during the ZIKV outbreak, after accounting for misclassification of GBS; ^c^ Proportion of false positive cases of GBS

## Discussion

Our findings suggest surveillance systems for ZIKV, microcephaly, and GBS in Latin America have a limited capacity to detect outbreaks. ZIKV outbreaks were detectable only through serological surveillance. Outbreaks of all and severe microcephaly were detected only when ZIKV infection increased the frequency of these conditions at least two and 24 times, respectively. Outbreaks of GBS were detectable only when GBS risk was at least eight times higher among ZIKV infected individuals. Finally, under most scenarios, cases of ZIKV infection, microcephaly, and GBS were more likely false positive than true positive cases.

While judging these findings, one should carefully consider the validity of study assumptions.

### ZIKV infection

We assumed a background risk of ZIKV infection of 10% and a doubling of the risk during an outbreak. A 10% risk corresponds to about half the risk in Brazil [41] and Puerto Rico [42]. A doubling of the risk is also consistent with a lowest limit of 10%, a most likely value of 25%, and an interquartile range of 19 to 33% suggested by Ellington et al. in a review of existing data [43]. Higher levels of baseline ZIKV infection risk would lower the chances of detecting an outbreak, due to a smaller pool of susceptible individuals [6, 44].

Values of sensitivity and specificity of ZIKV infection surveillance case definition were derived from the Yap Island study [11]. However, they are valid in Latin America, regardless of differences in prevalence, because sensitivity applies only to cases and specificity applies only to non-cases of a disease. The specificity of case definition for regular surveillance was high (95%), because it was applied to a self-selected sample of individuals who got medical care. This “pre-screening” improves specificity by increasing the prevalence of ZIKV infection among health care seekers. In contrast, the sensitivity was too low (2%). This is not surprising, because the definition only catches the 37.7% of all cases that are symptomatic [11] and only 11.5% of symptomatic cases seek medical care [45, 46]. Thus, the upper limit of sensitivity is 4.3%.

Due to the poor accuracy of ZIKV infection case definition, regular surveillance could show a decrease in incidence, even if the incidence has increased several fold. This could explain why incidence estimates from surveillance data has been orders of magnitude lower than those from serologic studies [11, 45], simulation studies [41, 47], and previous experience with viruses transmitted by the same vectors [43]. Also, this suggests regular ZIKV infection surveillance may be of little public health benefit.

Serologic surveillance seems the best option for ZIKV infection surveillance in Latin America. If serologic tests used in the Yap Island study were used, outbreaks would be detectable, though most cases would still be false positive. Yearly serologic surveys offer important advantages, compared to regular surveillance. They reduce the PFP; reflect trends in the whole population, instead of just health care seekers; provide needed data on background herd immunity [44]; and generate diagnostic accuracy data useful to correct risk estimates from regular surveillance [7]. Surveys could be conducted in random samples of a few hundred individuals (Additional file 1, item 12). Whether or not surveys are more cost-effective than regular surveillance is a moot point, since outbreaks cannot be detected using the latter. Well-planned serologic testing in random or haphazard samples of patients attending sentinel clinics could have similar benefits.

Unfortunately, better tests are needed for accurate diagnosis of ZIKV infection [48]. Moreover, the PFP cases would increase and outbreaks will be harder to detect if the Centers for Diseases Control’s (CDC) new guidelines to interpret serologic tests are used, as they improve sensitivity, in detriment of specificity [49].

In view of the uncertainty regarding the etiological role of ZIKV infection [5, 50–52], and the high PFP cases of ZIKV infection, even with serological testing, we should carefully ponder whether providing possibly infected patients with advice on conception attempts, changes in sexual behavior, and pregnancy outcomes is beneficial, cost-effective, or even ethical [53].

### Microcephaly

Due to the lack of published data, we estimated the accuracy of the diagnosis of microcephaly in normal weight newborn by simulation. Large studies of child growth standards [16, 54], support our assumption of a normal distribution of HC values, and TEM values used for simulation came from previous studies [18, 20].

Current surveillance systems target “congenital syndrome associated with Zika virus infection”, instead of all and severe microcephaly [1, 8, 9]. We focused on microcephaly because this is the main component of the postulated syndrome and it was possible to assess the accuracy if its diagnosis. Moreover, most components of the proposed syndrome are common findings in newborns with microcephaly of genetic and infectious origins [55, 56], and there is no evidence they cluster more frequently than expected in newborn from ZIKV-infected mothers [57]. More important, attributing all cases of microcephaly to ZIKV makes little sense. Indeed, about 50% of severe cases have a genetic etiology [14, 58], and 44 to 59% of non-severe cases could be due to low birth weight (Additional file 1, item 12). Moreover, some 10 to 33% of all cases should have maternal ZIKV infection, based on the risk of ZIKV infection [41–43], even if there is no association between the two conditions.

In spite of striking differences in prevalence, etiology, prognosis, and management [55, 59], current surveillance systems do not differentiate between severe and non-severe microcephaly [1, 8, 60, 61]. Therefore, surveillance data are of limited value for characterizing disease burden, making decisions about case management, and developing and allocating health resources.

Outbreaks of all microcephaly during a ZIKV infection outbreak will be detectable through regular surveillance if the risk of microcephaly is at least twice higher among infected mothers, but outbreaks of severe microcephaly would be virtually undetectable. Moreover, the PFP for microcephaly will be high, even if maternal ZIKV infection increased microcephaly prevalence by at least 15 times. Such a high prevalence ratio is very unlikely, since evidence from observational and ecological studies in Brazil and Colombia does not support an increased risk of microcephaly in newborn of ZIKV-infected mothers [5, 57].

It is uncertain whether adding non-severe microcephaly to the traditional surveillance of preterm birth and low-birth-weight [14] is justified in terms of its incremental cost-effectiveness to prevent perinatal and infant mortality. Indeed, half the cases of non-severe microcephaly may be due to low-birth-weight, and postnatal nutritional interventions is the cornerstone for the management of both conditions [62–65]. On the other hand, it is unlikely low-income countries will keep severe microcephaly as a public health priority, in view of the high impact of competing causes of perinatal and infant mortality, such as low birth weight, preterm birth, and neonatal asphyxia. Therefore, strengthening existing perinatal surveillance hospital networks may be the best option for surveillance of severe microcephaly [66, 67].

### Guillain-Barré syndrome

We used a GBS incidence of 2/100,000 in our analysis, which is higher than that in seven Latin American countries (1.41/100,000; Additional file 1, item 13) [3]. In addition, we evaluated scenarios where the risk of GBS among ZIKV-infected individuals increased five and 10 times. Such large increases in risk are unrealistic, since evidence from published studies [47, 68] do not support a causal ZIKV-GBS link [5, 50]. In addition, we used all published data to estimate the accuracy of a GBS diagnosis and the incidence of non-diabetic non-GBS peripheral neuropathy. Although these estimates may not strictly apply to Latin America, we believe they are robust enough for an informative assessment of GBS surveillance.

GBS surveillance data could be useful for health planning and program evaluation, but are of little use for outbreak control. In Latin America, GBS surveillance is compounded by the limited or non-existing capacity to perform motor nerve conduction tests, and to draw and analyze cerebrospinal fluid samples for cell count and protein concentration in most clinics. Without these tests, most GBS cases would have Brighton certainty levels ≤3, and a definite diagnosis may not be made until verifying the polyneuropathy was transient, something that could take several weeks [22, 69]. This compromises timely/accurate reporting of GBS cases. Thus, GBS surveillance may add little value to the analysis of data from existing sources [1, 2].

Assuming ongoing GBS surveillance is devoid of potential harm may be risky. As shown in this assessment, surveillance could result in large numbers of false positive cases. Premature declarations of GBS as a complication of ZIKV infection [1, 2], may contribute to misdiagnosing GBS-mimics as GBS cases. This could alter the distribution of scarce health resources in the region, distort health priorities and planning, increase the cost of surveillance activities, and add unnecessary testing, treatment, and morbidity in false positive GBS cases [22].

## Conclusions

Our findings suggest it is unlikely that outbreaks of ZIKV infection and putative related outcomes in Latin America will be detected through surveillance systems based on current guidelines [1, 2]. In consequence, it is unlikely these systems would be useful in detecting and curtailing impending or ongoing outbreaks, quantifying burden of disease, identifying factors driving risk, assessing the effectiveness of control measures, or improving clinical care.

In spite of uncertainty in some assumptions and parameters, we believe our findings are sufficiently robust to inform public health policies that, so far, seem mostly supported by questionable causal links and hopes of potential benefits. Policies in response to the ZIKV outbreaks were timely implemented, in a context of limited knowledge about causal links. Unfortunately, knowledge and data about non-causal issues, such as disease burden, diagnostic accuracy, and cost-effectiveness of potential interventions were given little weight when formulating surveillance guidelines. Though they are undoubtedly intended to improve and save lives, ZIKV public health policies should be based on previous experience and scientific knowledge, lest they become irrelevant and harmful for those they meant to protect [70].

## Additional file

Additional file 1: Appendix-Zika Surveillance-BMC PublicHealth. (PDF 844 kb)

## Abbreviations

CDC: Centers for Diseases Control
ELISA: Enzyme-Linked Immunosorbent Assay
EOCR: Expected observed case ratio
EORR: Expected observed risk ratio
GBS: Guillain-Barré syndrome
HC: Head circumference
HRI_Z → G_: Hypothetical relative increase in risk of GBS in Zika virus infected individuals
HRI_Z → M_: Hypothetical relative increase in risk of microcephaly among Zika virus infected individuals
IgM: Immunoglobulin M
NZ_(−)_: Number of ZIKV non-infected individuals during the outbreak
NZ_(+)_: Numbers of ZIKV infected individuals during the outbreak
PFP: Proportion of false positive cases
RG_0_: Baseline risk of Guillain-Barré syndrome
RRZ_0 → 1_: Hypothetical relative increase in the risk of Zika virus infection during the outbreak period
RT-PCR: Reverse transcription polymerase chain reaction
RZ_0_: Risk of Zika virus infection during the non-outbreak period
RZ_1_: Risk of Zika virus infection during the outbreak period
SD: Standard deviation
TEM: Technical error of measurements
ZIKV: Zika virus
