# Supplementary material for: An assessment of public health surveillance of Zika virus infection and potentially associated outcomes in Latin America
Source: BMC Public Health. 2018 May 24;18:656. doi: 10.1186/s12889-018-5566-7 (PMC5968501; doi:10.1186/s12889-018-5566-7)
Supplement: Supplementary file 1 — Appendix-Zika Surveillance-BMC PublicHealth. (PDF 844 kb) [file 12889_2018_5566_MOESM1_ESM.pdf]

Manuscript Title: An assessment of public health surveillance of Zika virus infection and potentially associated outcomes in Latin America.

Journal: BMC Public Health

Appendix

/*===*/

/*1.0 Overall methodological approach */

Figure 1. Overall approach for the comparison of the risk of Zika virus (ZIKV) infection, newborn microcephaly, and Guillain-Barré syndrome (GBS) during ZIKV infection outbreak and non-outbreak period.

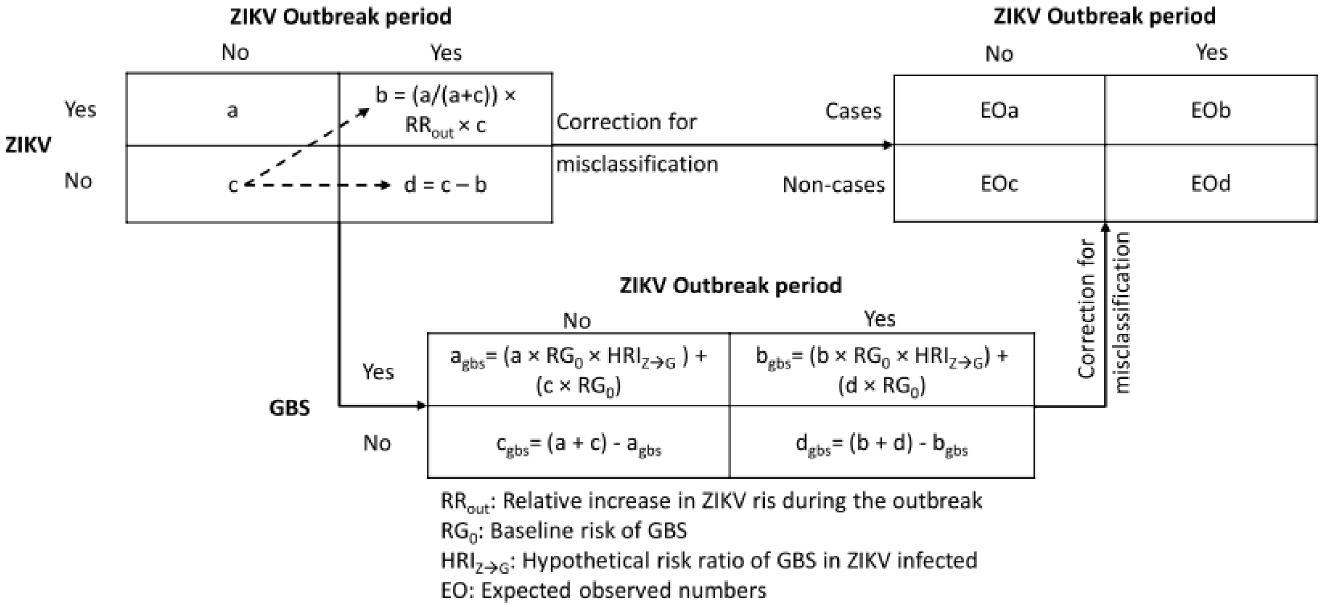

In the first table (top left), which corresponds to cases of ZIKV:

a = ZIKV cases during the non-outbreak period

c = ZIKV non-cases during the non-outbreak (i.e. RZ_0=***a***/(***a***+***c***) is the baseline risk of ZIKV)

b = ZIKV cases during the outbreak period. This corresponds to risk of ZIKV during the outbreak ($RZ_1 = RZ_0 \times 2$) times ***c***. While calculating ***b*** only ***c*** is taken into account because only they are susceptible to ZIKV during the outbreak period. Also, $RZ_1 = RZ_0 \times 2$, because we assume the risk doubles during the outbreak.

d = ***c*** – ***b***, because only ***b*** developed ZIKV during the outbreak.

In the second table (down), which corresponds to GBS cases:

a_{GBS} = GBS cases during the non-outbreak period. It includes cases that happened among individuals with ZIKV (***a*** in the first table) plus cases among individuals without ZIKV (***c*** in first table). Cell ***a*** contributes ***a*** times the baseline risk of GBS (RG_0) times the increase in baseline risk due to ZIKV

infection ($HRI_{Z\rightarrow G}$). Cell ***c*** only contributes ***c*** times the baseline risk of GBS, because among these individuals the risk of GBS has not changed.

c_{GBS} = (***a***+***c***) - a_{GBS}. That is the total number of individuals during the non-outbreak period (***a*** + ***b***) minus the total number of GBS cases during the non-outbreak period (a_{GBS})

b_{GBS} = GBS cases during the outbreak period. This includes cases in individuals with ZIKV (***b*** from first table) plus cases in individuals without ZIKV (***d*** from first table). The cell ***b*** contributes ***b*** times the baseline risk of GBS (RG_0) times the increase in baseline risk of GBS due to ZIKV infection ($HRI_{Z\rightarrow G}$). Cell ***d*** only contributes ***d*** times the baseline risk of GBS (RG_0), because among these individuals the risk of GBS has not changed.

d_{GBS} = (***b***+***d***) – b_{GBS}. That is the total number of individuals during the outbreak period (***b*** + ***d***) minus the total number of GBS cases during the outbreak period (d_{GBS}).

/*==*/

/*2.0 Estimating the sensitivity and specificity of the Zika virus infection case surveillance definition */

The surveillance case definition used in Latin America closely follows the one proposed by the Pan American Health Organization (PAHO): "Patient with rash with at least two or more of the following signs or symptoms: fever, usually <38.5 °C, conjunctivitis, arthralgia, myalgia, and peri-articular edema" [1]. This definition resembles the one used by Duffy et al. [2] in a study of an outbreak on Yap Island, Micronesia. Duffy et al. reviewed medical records and conducted prospective surveillance in all health centers to identify suspected ZIKV infection cases. They found 185 patients suspected cases, and classified 108 as definite or probable, based on ELISA tests for IgM antibodies and RT-PCR for ZIKV and dengue virus. They also conducted a serological survey in a random sample of the population and found an infection risk of 74.3% in 7931 Yap Island residents. We used those figures to estimate the sensitivity and the specificity of the case definition used for surveillance in three different scenarios.

For regular surveillance, which is similar to Duffy et al.'s study,[2] in the sense that the system detected only cases requesting medical care, sensitivity was estimated as the detection probability (108/7301) divided by the prevalence of ZIKV and specificity as 1 minus the false referral probability (77/7301) divided by 1 minus the prevalence of ZIKV (96%) [3, 4]. The resulting sensitivity and specificity were 2% and 96%, respectively.

For enhanced surveillance, we assumed the detection probability was five times higher in Latin America than in Yap Island [2]. In this case, specificity was 79.7% and sensitivity was 9.8%.

For serological surveillance, we assumed all suspected cases of ZIKV infection were detected and evaluated with the same laboratory tests used in the survey component of the Yap Island study [2]. In that study, 414 individuals had serologic evidence of recent ZIKV infection, 156 of whom reported an illness that met the definition of suspected ZIKV infection and 143 had no serologic evidence of ZIKV, 116 of whom did not reported an illness meeting the case definition. This corresponds to a case definition specificity of 81.1% and sensitivity of 37.7%

/*===*/

/*3.0 Estimating the sensitivity and specificity of head circumference measurements in the diagnosis of newborn microcephaly. Estimated values were used to account for misclassification of microcephaly. Estimates of the technical error of measurement (TEM) of head circumference were taken from de Onis [5] and from Bhushan et al. [6].*/

```
set more off
clear
capture program drop mfpr
qui program define mfpr
clear
foreach i in 0.1 /*0.2 0.25*/ {
```

```
qui set obs 1000000
qui set seed 7102127
qui gen hc=34.2 + 1.2 * invnorm(uniform())  /*Generates distribution of head circumference*/
qui sum hc
qui gen cut2sd=r(mean)-(2*r(sd))                /*Cut point defining all microcephaly*/
qui gen cut3sd=r(mean)-(3*r(sd))                /*Cut point defining severe microcephaly*/
qui gen micro2sd=hc<cut2sd                      /*True status for all microcephaly 0=No, 1=Yes*/
qui gen micro3sd=hc<cut3sd                     /*True status for severe microcephaly 0=No, 1=Yes*/
qui gen m2sderr=0

/*Sensitivity and specificity for intraobserver technical error of measurement: 0.24 cm*/
qui gen tem24=0 + 0.24 * invnorm(uniform())
qui gen hce24=hc+tem24
qui sum hce24
qui gen m2err24=hce24<cut2sd                 /*hc<c2err24*; True status for all microcephaly 0=No, 1=Yes*/
qui gen m3err24=hce24<cut3sd                 /*hc<c3err24; True status for severe microcephaly 0=No, 1=Yes*/
dis as text _col(2) _dup(90)"_"
dis as text _col(2) "Sensitivity and specificity of the diagnosis of microcephaly by level of technical error"
dis as text _col(2) "of measurement"
dis as text _col(2) _dup(90)"_"
dis as text _col(2) "Intraobserver TEM=0.24 (Onis et al.)"
qui diagt micro2sd m2err24
dis as text   _col(40) "Sensitivity"  _col(55) "Specificity"
dis as text _col(10) "All microcephaly"     _col(40) as res (r(sens)/100)  _col(55) as res (r(spec)/100)
qui diagt micro3sd m3err24
dis as text _col(10) "Severe microcephaly" _col(40) as res (r(sens)/100)  _col(55) as res (r(spec)/100)
dis as text _col(2) _dup(90)"-"

/*Sensitivity and specificity for interobserver technical measurement error: 0.42 cm*/
qui gen tem42=0 + 0.42 * invnorm(uniform())
qui gen hce42=hc + tem42
qui sum hce42
qui gen m2err42=hce42<cut2sd  /*hc<c2err42*/
                    /*True status for all microcephaly 0=No, 1=Yes*/
qui gen m3err42=hce42<cut3sd  /*hc<c3err42*/
                    /*True status for severe microcephaly 0=No, 1=Yes*/
dis as text _col(2) "Interobserver TEM=0.42 (Onis et al.)"
qui diagt micro2sd m2err42
dis as text _col(40) "Sensitivity"  _col(55) "Specificity"
dis as text _col(10) "All microcephaly"     _col(40) as res (r(sens)/100)  _col(55) as res (r(spec)/100)
qui diagt micro3sd m3err42
dis as text _col(10) "Severe microcephaly" _col(40) as res (r(sens)/100)  _col(55) as res (r(spec)/100)
dis as text _col(2) _dup(90)"_"

/*Sensitivity and specificity for interobserver technical measurement error: 0.71 cm*/
qui gen tem71=0 + 0.71 * invnorm(uniform())
qui gen hce71=hc + tem71
qui sum hce71
qui gen m2err71=hce71<cut2sd  /*hc<c2err42; True status for all microcephaly 0=No, 1=Yes*/
qui gen m3err71=hce71<cut3sd  /*hc<c3err42; True status for severe microcephaly 0=No, 1=Yes*/
```

```
dis as text _col(2) "Interobserver TEM=0.71 (Bhushan et al.) "
qui diagt micro2sd m2err71
dis as text _col(40) "Sensitivity"  _col(55) "Specificity"
dis as text _col(10) "All microcephaly"     _col(40) as res 0.9125  _col(55) as res 0.9723  _col(6do "appendix"
8) as text  "(Reported by Bhushan et al.)"
qui diagt micro3sd m3err71
dis as text _col(10) "Severe microcephaly" _col(40) as res (r(sens)/100)  _col(55) as res (r(spec)/100)
dis as text _col(2) _dup(90)"_"
}
end
mfpr

/*=========================================================================================*/
/*4.0 Estimating the sensitivity of Brighton certainty level ≤3 to diagnose Guillain-Barré syndrome [7-9]. */
scalar truenegatives=(385+22+339)    /*Data from references: [7-9] */
scalar totalcases=(494+29+386)
qui cii proportion totalcases truenegatives
dis as text _col(2) "Sensitivity of Brighton criteria, level>=3: "  as res _col(30) %4.2f r(proportion)*100 "% ("
%4.2f r(lb)*100  ", " %4.2f r(ub)*100 ")"

/*=========================================================================================*/
/*5.0 Estimating the incidence of non-diabetic non-GBS peripheral neuropathy, using data from references:
[10-20]*/
set more off
clear
input ord group num str20 diagnosis str20 location str20 author year csmal rmal csfem rfem csall rall lbrall
ubrall str20 comment
1  1 1    "Bell's palsy"  "Minnesota, USA" "Katusic, 1986" 1986  85  22.8  121 26.9  206       25.0      .            . ""
2  1 2    "Bell's palsy"  "Texas, USA" "Brandenburg, 1993" 1993  87  23.5  134 32.7  221      29.1      .            . ""
3  1 3    "Bell's palsy"  "Ehime, Japan" "Yanagihara, 1988" 1988  .   .       .          .  459      30.0      .            . ""
4  2 1    "Cerv radiculopathy" "Minnesota" "Radhakrishnan, 1994" 1994 332 104.3 229 58.3  560   79.0  .   . ""
5  3 1    "Carpal tunnel syn"  "Minnesota"  "Stevens, 1988" 1988  .   .   .   .         1016   99.0        .            . ""
6  3 2    "Carpal tunnel syn"  "Minnesota"  "Vessey, 1990"   1990  .   .  154  81.7  154   81.7  .                   . ""
7  3 3    "Carpal tunnel syn"   "UK"  "Rowlands, 2002"  2002  .    .   .   .  2473   20.2        .            .        ""
8  4 1    "Brachial plexus neur"    "Minnesota"     "Beghi, 1985"    1985  .   .   .   .  11   1.6    .          .        ""
9  5 1    "Distal Symetric Neur"    "Italy"    "Baldereschi, 2007"   2007  .     .    .      .  63   576.0 426.0 726.0
          "Non-diabetic (65-84 yrs)"
10 6 1  "Polyneuropathy"  "Minnesota"   "Beghi, 1985"    1985  . .          .          .    310   46.2      .            .
          "Polyneuropathy (other than BPN)"
11 6 3  "Polyneuropathy" "South-Rogaland, Norway" "Rudolph, 2007" 2007  .    . .        .  36     11.9    .   .
          "Non-diabetic/non-GBS (31-91 yrs)"
end
capture program drop pnrate
quietly program define pnrate
gen popall=int(100000*csall/rall)
format diagnosis location %-20s
format r* lb* ub* %3.1fc
preserve
mkmat ord group num csall popall, matrix(R)
//matrix list R
```

```
qui replace ord=_n
local max=_N
forvalues i=1/`max' {
	local num=R[`i',4]
	local den=R[`i',5]
	qui cii proportion `den' `num'
	qui replace lbrall=r(lb)*100000 if ord==`i' & lbrall==.
	qui replace ubrall=r(ub)*100000 if ord==`i' & ubrall==.
}
//list csall pop *rall
qui metan rall lbrall ubrall, random by(diagnosis) label(namevar=author, yearid=year) nograph
dis as text _col(2) "Incidence of non-diabetic, non-GBS peripheral neuropaty (/100,000):"
dis as res _col(2) %4.2f r(ES) "  (" %4.2f r(ci_low) ", " %4.2f r(ci_upp) ")"
restore
end
pnrate

/*==========================================================================================*/
```

/*6.0 Using bias analysis to estimate the impact of sensitivity and specificity of surveillance case definition on the detection of an outbreak. See Lash et al. for formulae [21]. "*/

```
capture program drop chk
quietly program define chk
clear
qui set obs 1
/*Setting scalar values*/
scalar hrr=`1'
scalar sensE1=`2'
scalar sensE0=`3'
scalar specE1=`4'
scalar specE0=`5'
scalar pop=1000000
scalar b=pop*0.10                 /*b: non-exposed cases*/
scalar d=pop-b                    /*d: non-exposed non-cases*/
scalar a=(pop-b)*0.10*`1'         /*a: exposed cases*/
scalar c=(pop-b)-a                /*c: expose non-cases*/

scalar oa=(a*sensE1) + (c*(1-specE1))
scalar ob=(b*sensE0) + (d*(1-specE0))
scalar oc=(c*specE1) + (a*(1-sensE1))
scalar od=(d*specE0) + (b*(1-sensE0))
scalar ppv=(1-(((c*(1-specE1)) + (d*(1-specE0)))/(oa+ob)))*100  /*This is the positive predictive value*/
end

/*Scenario: Regular surveillance, hospital case detection*/
capture program drop dochk1
qui program define dochk1
tempname memhold
tempfile q8
postfile `memhold' str10 type1 double(hrr spec1 sens1 eorr1 eocr1 rrpvalue1 ppv1) using `q8'
scalar sens1=0.01965963
```

```
scalar spec1=0.9594205
	forvalues j = 1(0.1)9 {
		foreach i in 1  {
		chk `j' sens1 sens1 spec1 spec1
		local a1=round(oa,1)
		local b1=round(ob,1)
		local c1=round(oc,1)
		local d1=round(od,1)
		qui csi `a1' `b1' `c1' `d1'
		qui gen eorr1=r(rr)
		qui gen rrpvalue1=r(p)
		qui gen eocr1=`a1'/`b1'
		qui gen ppv1=ppv
		gen type1="Regular"
		post `memhold' (type1) (hrr) (spec1) (sens1) (eorr1) (eocr1) (rrpvalue1) (ppv1)
		}
	}
postclose `memhold'
use `q8', clear
scalar z1=(0 - (0+(1.96*1))/1)
gen pcut=(1-normal(abs(z1)))*2
label var pcut "Outbreak detection cut-point"
label var hrr "Hypotethical risk ratio"
//label var rrpvalue1 "Sensitivity: 1.96%"
end
dochk1

/*Scenario: Enhanced surveillance; case detection probability increased 5-fold*/
capture program drop dochk2
qui program define dochk2
tempname memhold
tempfile q8
postfile `memhold' str10 type2 double(hrr spec2 sens2 eorr2 eocr2 rrpvalue2 ppv2) using `q8'
scalar sens2=0.09829817
scalar spec2=0.79710251
	forvalues j = 1(0.1)9 {
		foreach i in 1  {
		chk `j' sens2 sens2 spec2 spec2
		local a2=round(oa,1)
		local b2=round(ob,1)
		local c2=round(oc,1)
		local d2=round(od,1)
		qui csi `a2' `b2' `c2' `d2'
		qui gen eorr2=r(rr)
		qui gen rrpvalue2=r(p)
		qui gen eocr2=`a2'/`b2'
		qui gen ppv2=ppv
		gen type2="Enhanced"
		post `memhold' (type2) (hrr) (spec2) (sens2) (eorr2) (eocr2) (rrpvalue2) (ppv2)
		}
```

```
        }
postclose `memhold'
use `q8', clear
scalar z1=(0 - (0+(1.96*1))/1)
gen pcut=(1-normal(abs(z1)))*2
label var pcut "Outbreak detection cut-point"
label var hrr "Hypotethical risk ratio"
end
dochk2

/*Scenario: Serological surveillance; population survey with serological testing*/
capture program drop dochk3
qui program define dochk3
tempname memhold
tempfile q8
scalar sens3=0.3768
scalar spec3=0.8111
postfile `memhold' str10 type3 double(hrr spec3 sens3 eorr3 eocr3 rrpvalue3 ppv3) using `q8'
        forvalues j = 1(0.1)9 {
                foreach i in 1  {
                chk `j' sens3 sens3 spec3 spec3
                local a3=round(oa,1)
                local b3=round(ob,1)
                local c3=round(oc,1)
                local d3=round(od,1)
                qui csi `a3' `b3' `c3' `d3'
                qui gen eorr3=r(rr)
                qui gen rrpvalue3=r(p)
                qui gen eocr3=`a3'/`b3'
                qui gen ppv3=ppv
                gen type3="Serologic"
                post `memhold' (type3) (hrr) (spec3) (sens3) (eorr3) (eocr3) (rrpvalue3) (ppv3)
                }
        }
postclose `memhold'
use `q8', clear
scalar z1=(0 - (0+(1.96*1))/1)
gen pcut=(1-normal(abs(z1)))*2
label var pcut "Outbreak detection cut-point"
label var hrr "Hypotethical risk ratio"
end
dochk3

capture program drop minrr
qui program define minrr
dochk1
qui save zikv1, replace
dochk2
qui save zikv2, replace
dochk3
```

```
qui save zikv3, replace
use zikv1, clear
qui merge 1:1 hrr using zikv2
qui drop _merge
qui merge 1:1 hrr using zikv3
qui drop _merge
dis _n
dis as text _col(2) "Minimum hypothetical risk ratio to detect an outbreak of ZIKV infection,"
dis as text _col(2) "accounting for different levels of sensitivity and specificity."
dis as text _col(2) _dup(80)"_"
dis _col(2) as text "Surveillance"  _col(20) "Hypothetical"            _col(40) "Observed"
            _col(60) "P-value" _col(70) "Case ratio"
dis _col(5) as text                                                         _col(20) "risk ratio"                    _col(40) "risk ratio"
dis as text _col(2) _dup(80)"_"
foreach i in 1 2 3 {
            qui sum hrr if rrpvalue`i'<pcut  & eorr`i'>=1
            scalar minhrr`i'=r(min)
            qui sum eorr`i' if rrpvalue`i'<pcut  & eorr`i'>=1
            scalar minorr`i'=r(min)
            qui sum rrpvalue`i' if rrpvalue`i'<pcut  & eorr`i'>=1
            scalar maxp=r(max)
            qui sum eocr`i' if rrpvalue`i'<pcut  & eorr`i'>=1
            scalar maxcr`i'=r(min)
            dis as res _col(5) type`i'              _col(20) %8.6f minhrr`i'  _col(40) %8.6f minorr`i'  _col(60) %8.6f maxp
_col(70) %6.4f maxcr`i'
}
dis as text _col(2) _dup(80)"_"
end
minrr
capture program drop dolist
qui program define dolist
gen id=_n
dis _n
dis as text _col(2) "Table 1. Observed expected risk ratios (EORR) and case ratios (EOCR) of Zika virus (ZIKV)"
dis as text _col(2) "infection during an outbreak period by type of surveillance approach, hypothetical relative"
dis as text _col(2) "increase (HRR) in risk of ZIKV infection during the outbreak, and by level of sensitivity"
dis as text _col(2) "and specificity of the approach to detect cases of infection."
dis as text _col(2) _dup(90)"_"
dis as text _col(2) "HRR" _col(12) "Sens" _col(22) "Spec"  _col(32) "EORR"  _col(42) "EOCR"  _col(52) "PPV"
dis as text _col(2) _dup(90)"_"
dis as text _col(2) "Regular surveillance"
foreach var in hrr sens1 spec1 eorr1 eocr1 ppv1 {
            qui sum `var' if id==1
            scalar m`var'=r(mean)
            }
dis as res _col(2) as res mhrr _col(12) %4.2f (msens1)*100 _col(22) %4.2f (mspec1 )*100  _col(32) %4.2f
meorr1 _col(42) %4.2f meocr1 _col(52) %4.2f mppv1
foreach var in hrr sens1 spec1 eorr1 eocr1 ppv1 {
            qui sum `var' if id==11
            scalar m`var'=r(mean)
```

```
            }
dis as res _col(2) as res mhrr _col(12) %4.2f (msens1)*100 _col(22) %4.2f (mspec1 )*100  _col(32) %4.2f
meorr1 _col(42) %4.2f meocr1 _col(52) %4.2f mppv1
foreach var in hrr sens1 spec1 eorr1 eocr1 ppv1 {
            qui sum `var' if id==41
            scalar m`var'=r(mean)
            }
dis as res _col(2) as res mhrr _col(12) %4.2f (msens1)*100 _col(22) %4.2f (mspec1 )*100  _col(32) %4.2f
meorr1 _col(42) %4.2f meocr1 _col(52) %4.2f mppv1
foreach var in hrr sens1 spec1 eorr1 eocr1 ppv1 {
            qui sum `var' if id==71
            scalar m`var'=r(mean)
            }
dis as res _col(2) as res mhrr _col(12) %4.2f (msens1)*100 _col(22) %4.2f (mspec1 )*100  _col(32) %4.2f
meorr1 _col(42) %4.2f meocr1 _col(52)  %4.2f mppv1
dis as text _col(2) _dup(90)"-"
dis as text _col(2) "Enhanced surveillance"
foreach var in hrr sens2 spec2 eorr2 eocr2 ppv2 {
            qui sum `var' if id==1
            scalar m`var'=r(mean)
            }
dis as res _col(2) as res mhrr _col(12) %4.2f (msens2)*100 _col(22) %4.2f (mspec2)*100  _col(32) %4.2f
meorr2 _col(42) %4.2f meocr2 _col(52) %4.2f mppv2
foreach var in hrr sens2 spec2 eorr2 eocr2 ppv2 {
            qui sum `var' if id==11
            scalar m`var'=r(mean)
            }
dis as res _col(2) as res mhrr _col(12) %4.2f (msens2)*100 _col(22) %4.2f (mspec2)*100  _col(32) %4.2f
meorr2 _col(42) %4.2f meocr2 _col(52) %4.2f mppv2
foreach var in hrr sens2 spec2 eorr2 eocr2 ppv2 {
            qui sum `var' if id==41
            scalar m`var'=r(mean)
            }
dis as res _col(2) as res mhrr _col(12) %4.2f (msens2)*100 _col(22) %4.2f (mspec2)*100  _col(32) %4.2f
meorr2 _col(42) %4.2f meocr2 _col(52) %4.2f mppv2
foreach var in hrr sens2 spec2 eorr2 eocr2 ppv2 {
            qui sum `var' if id==71
            scalar m`var'=r(mean)
            }
dis as res _col(2) as res mhrr _col(12) %4.2f (msens2)*100 _col(22) %4.2f (mspec2)*100  _col(32) %4.2f
meorr2 _col(42) %4.2f meocr2 _col(52)  %4.2f mppv2
dis as text _col(2) _dup(90)"-"
dis as text _col(2) "Serologic surveillance"
foreach var in hrr sens3 spec3 eorr3 eocr3 ppv3 {
            qui sum `var' if id==1
            scalar m`var'=r(mean)
            }
dis as res _col(2) as res mhrr _col(12) %4.2f (msens3)*100 _col(22) %4.2f (mspec3)*100  _col(32) %4.2f
meorr3 _col(42) %4.2f meocr3 _col(52) %4.2f mppv3
foreach var in hrr sens3 spec3 eorr3 eocr3 ppv3 {
```

```
		qui sum `var' if id==11
		scalar m`var'=r(mean)
		}
dis as res _col(2) as res mhrr _col(12) %4.2f (msens3)*100 _col(22) %4.2f (mspec3)*100  _col(32) %4.2f
meorr3 _col(42) %4.2f meocr3 _col(52) %4.2f mppv3
foreach var in hrr sens3 spec3 eorr3 eocr3 ppv3 {
		qui sum `var' if id==41
		scalar m`var'=r(mean)
		}
dis as res _col(2) as res mhrr _col(12) %4.2f (msens3)*100 _col(22) %4.2f (mspec3)*100  _col(32) %4.2f
meorr3 _col(42) %4.2f meocr3 _col(52) %4.2f mppv3
foreach var in hrr sens3 spec3 eorr3 eocr3 ppv3 {
		qui sum `var' if id==71
		scalar m`var'=r(mean)
		}
dis as res _col(2) as res mhrr _col(12) %4.2f (msens3)*100 _col(22) %4.2f (mspec3)*100  _col(32) %4.2f
meorr3 _col(42) %4.2f meocr3 _col(52)  %4.2f mppv3
dis as text _col(2) _dup(90)"-"
end
dolist
```

/*===*/

/*7.0 Using bias analysis to estimate the impact of sensitivity and specificity of surveillance case definition on the detection of an outbreak of microcephaly during an outbreak of Zika virus infection. Also, estimating the minimum hypothetical relative increase in the risk of microcephaly among newborn of infected women that would result in the detection of an outbreak. For formulae, see Lash et al. [21].*/

```
capture program drop allmicro   /*Head circumference <2 SD*/
quietly program define allmicro
clear
qui set obs 1
scalar z2=(32-(32-(2*1.2)))/1.2
scalar m2sdrisk=(1-normal(abs(z2))) /*This is the risk of all microcephaly*/
scalar zrisk=0.10
scalar hrr=`1'				/*This is the increase in risk of microcephaly among ZIKV infected mothers*/
scalar sensE1=`2'
scalar sensE0=`3'
scalar specE1=`4'
scalar specE0=`5'
scalar pop=173000		/*Number of births if pop size equals median in LA: 10 million; background period*/
scalar e0z1m1=(pop*zrisk)*(m2sdrisk*`1')			/*Cell a: Exposure=0; Zika=1; micro=1*/
scalar e0z1m0=(pop*zrisk)-e0z1m1				/*Cell c: Exposure=0; Zika=1; micro=0*/
scalar e0z0m1=(pop*(1-zrisk))*(m2sdrisk)			/*Cell b: Exposure=0; Zika=0; micro=1*/
scalar e0z0m0=(pop*(1-zrisk))-e0z0m1				/*Cell d: Exposure=0; Zika=0; micro=0*/
scalar B=round((e0z1m1+e0z0m1),1)
scalar D=round((e0z1m0+e0z0m0),1)
scalar susceptible2=pop*(1-zrisk)
/*Outbreak period*/
scalar e1z1m1=(susceptible2*zrisk*2)*(m2sdrisk*`1')
scalar e1z1m0=(susceptible2*zrisk*2)- e1z1m1
scalar e1z0m1=(pop-(susceptible2*zrisk*2))*(m2sdrisk)
```

```
scalar e1z0m0=(pop-(susceptible2*zrisk*2))-e1z0m1
scalar A=round((e1z1m1+e1z0m1),1)
scalar C=round((e1z1m0+e1z0m0),1)

/*Obtaining expected observed number of events, based on sensitivity and specificity of diagnostic test*/
scalar oa=(A*sensE1) + (C*(1-specE1))
scalar ob=(B*(sensE0)) + (D*(1-specE0))
scalar oc=(C*specE1) + (A*(1-sensE1))
scalar od=(D*(specE0)) + (B*(1-sensE0))
scalar ppv=(1-(((C*(1-specE1)) + (D*(1-specE0)))/(oa+ob)))*100  /*Positive predictive value: 1-(FP/All positives)*/
scalar trueRR=(A/(A+C))/(B/(B+D))                          /*RR if there were no misclassification errors*/
end

capture program drop doallmicro
qui program define doallmicro
tempname memhold
tempfile q8
postfile `memhold' double(hrr rr_all_24 rrpvalue_all_24 cr_all_24 sens_all_24 spec_all_24) using `q8'
        forvalues j = 1(0.5)60 {
        allmicro `j'  .85098587   .85098587 .9942287 .9942287  /*Intraobserver TEM=0.24*/
        local a24=round(oa,1)
        local b24=round(ob,1)
        local c24=round(oc,1)
        local d24=round(od,1)
        qui csi `a24' `b24' `c24' `d24'
        qui gen rr_all_24=r(rr)
        qui gen rrpvalue_all_24=r(p)
        qui gen cr_all_24=`a24'/`b24'    /*case ratio is almost identical to prevalence ratio*/
        qui gen sens_all_24=.85098587
        qui gen spec_all_24=.9942287
        post `memhold' (hrr) (rr_all_24) (rrpvalue_all_24) (cr_all_24) (sens_all_24) (spec_all_24)
        }
postclose `memhold'
use `q8', clear
label var hrr "Hypotethical risk ratio"
dis hrr "  " rr_all_24 "   " rrpvalue_all_24
label var rrpvalue_all_24 "All microcephaly"
save t1, replace

tempname memhold
tempfile q8
postfile `memhold' double(hrr rr_all_42 rrpvalue_all_42 sens_all_42 spec_all_42) using `q8'
        forvalues j = 1(0.5)60 {
        allmicro `j'   .7756936   .7756936    .98779317      .98779317  /*Intraobserver TEM=0.42*/
        local a42=round(oa,1)
        local b42=round(ob,1)
        local c42=round(oc,1)
        local d42=round(od,1)
        qui csi `a42' `b42' `c42' `d42'
```

```
		qui gen rr_all_42=r(rr)
		qui gen rrpvalue_all_42=r(p)
		qui gen sens_all_42=.7756936
		qui gen spec_all_42=.98779317
		post `memhold' (hrr) (rr_all_42) (rrpvalue_all_42) (sens_all_42) (spec_all_42)
		}
postclose `memhold'
use `q8', clear
label var hrr "Hypotethical risk ratio"
dis hrr "  " rr_all_42 "   " rrpvalue_all_42
label var rrpvalue_all_42 "All microcephaly"
save t2, replace
end
doallmicro
qui merge 1:1 hrr using t1
drop _merge
save allmic, replace

capture program drop sevmicro
quietly program define sevmicro
clear
qui set obs 1
scalar z2=(32-(32-(3*1.2)))/1.2
scalar m3sdrisk=(1-normal(abs(z2))) /*This is the risk of severe microcephaly*/
scalar zrisk=0.10
scalar hrr=`1'				/*This is the increase in risk of microcephaly among ZIKV infected mothers*/
scalar sensE1=`2'
scalar sensE0=`3'
scalar specE1=`4'
scalar specE0=`5'
scalar pop=10000
/*Background period*/
scalar e0z1m1=(pop*zrisk)*(m3sdrisk*`1')			/*Cell a: Exposure=0; Zika=1; micro=1*/
scalar e0z1m0=(pop*zrisk)-e0z1m1				/*Cell c: Exposure=0; Zika=1; micro=0*/
scalar e0z0m1=(pop*(1-zrisk))*(m3sdrisk)			/*Cell b: Exposure=0; Zika=0; micro=1*/
scalar e0z0m0=(pop*(1-zrisk))-e0z0m1			/*Cell d: Exposure=0; Zika=0; micro=0*/
scalar B=round((e0z1m1+e0z0m1),1)
scalar D=round((e0z1m0+e0z0m0),1)
scalar susceptible2=pop*(1-zrisk)
/*Outbreak period*/
scalar e1z1m1=(susceptible2*zrisk*2)*(m3sdrisk*`1')
scalar e1z1m0=(susceptible2*zrisk*2)- e1z1m1
scalar e1z0m1=(pop-(susceptible2*zrisk*2))*(m3sdrisk)
scalar e1z0m0=(pop-(susceptible2*zrisk*2))-e1z0m1
scalar A=round((e1z1m1+e1z0m1),1)
scalar C=round((e1z1m0+e1z0m0),1)
/*Correcting for misclassification error*/
scalar oa=(A*sensE1) + (C*(1-specE1))
scalar ob=(B*sensE0) + (D*(1-specE0))
scalar oc=(C*specE1) + (A*(1-sensE1))
```

```
scalar od=(D*specE0) + (B*(1-sensE0))
scalar ppv=(1-(((C*(1-specE1)) + (D*(1-specE0)))/(oa+ob)))*100  /*This is the positive predictive value*/
scalar trueRR=(A/(A+C))/(B/(B+D))  /*RR if there were no misclassification errors*/
end

capture program drop dosevmicro
qui program define dosevmicro
tempname memhold
tempfile q8
postfile `memhold' double(hrr rr_sev_24 rrpvalue_sev_24 cr_sev_24 sens_sev_24 spec_sev_24) using `q8'
        forvalues j = 1(0.5)60 {
        sevmicro `j'   .81904762   .81904762  .99943423    .99943423  /*Intraobserver TEM=0.24*/
        local a24=round(oa,1)
        local b24=round(ob,1)
        local c24=round(oc,1)
        local d24=round(od,1)
        qui csi `a24' `b24' `c24' `d24'
        qui gen rr_sev_24=r(rr)
        qui gen rrpvalue_sev_24=r(p)
        qui gen cr_sev_24=`a24'/`b24'
        qui gen sens_sev_24=.81904762
        qui gen spec_sev_24=.99943423
        post `memhold' (hrr) (rr_sev_24) (rrpvalue_sev_24) (cr_sev_24) (sens_sev_24) (spec_sev_24)
        }
postclose `memhold'
use `q8', clear
label var hrr "Hypotethical risk ratio"
label var rrpvalue_sev_24 "Observed expected risk ratio%"
dis hrr "  " rr_sev_24 "   " rrpvalue_sev_24
scalar z1=(0 - (0+(1.96*1))/1)
gen pcut=(1-normal(abs(z1)))*2
label var pcut "Outbreak detection cut-point"
label var hrr "Hypotethical risk ratio"
label var rrpvalue_sev_24 "Severe microcephaly"
qui save t1, replace

tempname memhold
tempfile q8
postfile `memhold' double(hrr rr_sev_42 rrpvalue_sev_42 sens_sev_42 spec_sev_42) using `q8'
        forvalues j = 1(0.5)60 {
        sevmicro `j'  .74945055  .74945055  .9986842 .9986842  /*Interobserver TEM=0.42*/
        local a42=round(oa,1)
        local b42=round(ob,1)
        local c42=round(oc,1)
        local d42=round(od,1)
        qui csi `a42' `b42' `c42' `d42'
        qui gen rr_sev_42=r(rr)
        qui gen rrpvalue_sev_42=r(p)
        qui gen sens_sev_42=.74945055
        qui gen spec_sev_42=.9986842
```

```
            post `memhold' (hrr) (rr_sev_42) (rrpvalue_sev_42) (sens_sev_42) (spec_sev_42)
            }
postclose `memhold'
use `q8', clear
label var hrr "Hypotethical risk ratio"
label var rrpvalue_sev_42 "Observed expected risk ratio%"
dis hrr "  " rr_sev_42 "   " rrpvalue_sev_42
scalar z1=(0 - (0+(1.96*1))/1)
gen pcut=(1-normal(abs(z1)))*2
label var pcut "Outbreak detection cut-point"
label var hrr "Hypotethical risk ratio"
label var rrpvalue_sev_42 "Severe microcephaly"
end
dosevmicro
qui merge 1:1 hrr using t1
drop _merge
qui merge 1:1 hrr using allmic
drop _merge
qui save t2, replace

capture program drop doallmicro
qui program define doallmicro
tempname memhold
tempfile q8
postfile `memhold' double(hrr rr_all_71 rrpvalue_all_71 sens_all_71 spec_all_71) using `q8'
            forvalues j = 1(0.5)60 {
            allmicro `j' 0.9125 0.9125 0.9723 0.9723  /*Intraobserver TEM=0.71; sensitivity and specicifity as
                                                      reported in study*/
            local a24=round(oa,1)
            local b24=round(ob,1)
            local c24=round(oc,1)
            local d24=round(od,1)
            qui csi `a24' `b24' `c24' `d24'
            qui gen rr_all_71=r(rr)
            qui gen rrpvalue_all_71=r(p)
            qui gen sens_all_71=0.9125
            qui gen spec_all_71=0.9723
            post `memhold' (hrr) (rr_all_71) (rrpvalue_all_71) (sens_all_71) (spec_all_71)
            }
postclose `memhold'
use `q8', clear
label var hrr "Hypotethical risk ratio"
dis hrr "  " rr_all_71 "   " rrpvalue_all_71
label var rrpvalue_all_71 "All microcephaly"
end
doallmicro
qui merge 1:1 hrr using t2
drop _merge
save t3, replace
```

```
capture program drop dosevmicro
qui program define dosevmicro
tempname memhold
tempfile q8
postfile `memhold' double(hrr rr_sev_71 rrpvalue_sev_71 sens_sev_71 spec_sev_71) using `q8'
	forvalues j = 1(0.5)60 {
	sevmicro `j' .65934066 .65934066 .99594346  .99594346    /*Intraobserver TEM=0.71; sensitivity and
	                                                             specificity obtained by simulation*/
	local a24=round(oa,1)
	local b24=round(ob,1)
	local c24=round(oc,1)
	local d24=round(od,1)
	qui csi `a24' `b24' `c24' `d24'
	qui gen rr_sev_71=r(rr)
	qui gen rrpvalue_sev_71=r(p)
	qui gen sens_sev_71=.65934066
	qui gen spec_sev_71=.99594346
	post `memhold' (hrr) (rr_sev_71) (rrpvalue_sev_71) (sens_sev_71) (spec_sev_71)
	}
postclose `memhold'
use `q8', clear
label var hrr "Hypotethical risk ratio"
dis hrr "  " rr_sev_71 "   " rrpvalue_sev_71
label var rrpvalue_sev_71 "All microcephaly"
end
dosevmicro
qui merge 1:1 hrr using t3
drop _merge

capture program drop minrr
qui program define minrr
dis _n
dis as text _col(2) "Table 2. Minimum hypothetical risk ratio to detect an outbreak of microcephaly and
expected observed"
dis as text _col(2) "risk ratios if ZIKV risk doubles during an outbreak, correcting for sensitivity and specificity"
dis as text _col(2) "of microcephaly case detection"
dis as text _col(2) _dup(90)"_"
dis _col(2) as text _col(2) "Microcephaly" _col(20) "TEM*"  _col(30) "Sensitivity"  _col(45) "Specificity" _col(60)
"Hypothetical"   _col(80) "Observed"
dis _col(2) as text  _col(20) "(cm)"  _col(30) "%"   _col(45) "%"  _col(60) "risk ratio"  _col(80) "risk ratio"
dis as text _col(2) _dup(90)"_"
dis as text _col(2) "All"
foreach i in 24 42 71 {
	qui sum hrr if rrpvalue_all_`i'<pcut
	scalar minhrr_all_`i'=r(min)
	qui sum rr_all_`i' if rrpvalue_all_`i'<pcut
	scalar minorr_all_`i'=r(min)
	dis as res		_col(20) `i'/100 _col(30) %4.2f (sens_all_`i'*100) _col(45) %4.2f (spec_all_`i'*100)
_col(60) %4.2f minhrr_all_`i'  _col(80) %4.2f minorr_all_`i'
}
```

```
dis as text _col(2) _dup(90)"_"
dis as text _col(2) "Severe"
foreach i in 24 42 71 {
	qui sum hrr if rrpvalue_sev_`i'<pcut
	scalar minhrr_sev_`i'=r(min)
	qui sum rr_sev_`i' if rrpvalue_sev_`i'<pcut
	scalar minorr_sev_`i'=r(min)
	dis as res 	_col(20) `i'/100 _col(30) %4.2f (sens_sev_`i'*100) _col(45) %4.2f (spec_sev_`i'*100) 
_col(60) %4.2f minhrr_sev_`i'  _col(80) %4.2f minorr_sev_`i'
}
dis as text _col(2) _dup(90)"_"
dis as text _col(2) "* TEM: Technical error of head circumference measurements. TEM=0.71 came from 
Bhushan et al"
dis as text _col(2) "(J Clin Epidemiol Vol. 44 (10):1027-1035, 1991),but values of sensitivity and specificity"
dis as text _col(2) "for all microcephaly were taken directly from theirarticle, while those for severe were"
dis as text _col(2) "obtained by simulation. All other TEM values came from Onis (Acta Pediatrica, 2006;"
dis as text _col(2) "Suppl 450:38-46) and were used to estimate sensitivity and specificity by simulation."
end
minrr
```

```
/*===========================================================================================*/
```

/*8.0 Estimating the impact of sensitivity and specificity on the detection of an outbreak of all microcephaly associated with an outbreak of Zika virus infection. See Lash et al. [21]. */

```
/*8.1 All microcephaly*/
capture program drop allmicro
quietly program define allmicro
clear all
scalar z2=(32-(32-(2*1.2)))/1.2
scalar m2sdrisk=(1-normal(abs(z2))) /*This is the risk of all microcephaly*/
scalar zrisk=0.10
scalar hrr=`1'			/*This is the increase in risk of microcephaly among ZIKV infected mothers*/
scalar sensE1=`2'
scalar sensE0=`3'
scalar specE1=`4'
scalar specE0=`5'
scalar pop=173000		/*Number of births if population size equals median in LA: 10 million*/
/*Background period*/
scalar e0z1m1=(pop*zrisk)*(m2sdrisk*`1')		/*Cell a: Exposure=0; Zika=1; micro=1*/
scalar e0z1m0=(pop*zrisk)-e0z1m1			/*Cell c: Exposure=0; Zika=1; micro=0*/
scalar e0z0m1=(pop*(1-zrisk))*(m2sdrisk)		/*Cell b: Exposure=0; Zika=0; micro=1*/
scalar e0z0m0=(pop*(1-zrisk))-e0z0m1			/*Cell d: Exposure=0; Zika=0; micro=0*/
scalar B=round((e0z1m1+e0z0m1),1)
scalar D=round((e0z1m0+e0z0m0),1)
scalar susceptible2=pop*(1-zrisk)
/*Outbreak period*/
scalar e1z1m1=(susceptible2*zrisk*2)*(m2sdrisk*`1')
 scalar e1z1m0=(susceptible2*zrisk*2)- e1z1m1
scalar e1z0m1=(pop-(susceptible2*zrisk*2))*(m2sdrisk)
scalar e1z0m0=(pop-(susceptible2*zrisk*2))-e1z0m1
scalar A=round((e1z1m1+e1z0m1),1)
```

```
scalar C=round((e1z1m0+e1z0m0),1)
/*Obtaining expected observed number of events, based on sensitivity and specificity of diagnostic test*/
scalar oa=(A*sensE1) + (C*(1-specE1))
scalar ob=(B*(sensE0)) + (D*(1-specE0))
scalar oc=(C*specE1) + (A*(1-sensE1))
scalar od=(D*(specE0)) + (B*(1-sensE0))
scalar ppv=(1-(((C*(1-specE1)) + (D*(1-specE0)))/(oa+ob)))*100  /*This is the PPV: 1-(FP/All positives)*/
scalar trueRR=(A/(A+C))/(B/(B+D))  /*RR if there were no misclassification errors*/
end

capture program drop doallmicro
qui program define doallmicro
dis as text _col(2) "Table 3. Observed relative increase in prevalence of newborn all microcephaly and positive predictive"
dis as text _col(2) "values (PPV) in an outbreak as compared to a non-outbreak period of Zika virus (ZIKV) infection, by"
dis as text _col(2) "by hypothetical relative increase in the prevalence of microcephaly in newborn of ZIKV-infected mothers,"
dis as text _col(2)           "and by level of intra and inter-observer technical error of measurement (TEM) of head circumference."
dis as text _col(2) _dup(100)"_"
dis as text _col(2) "True risk" _col(15) "RR*"  _col(20) "Sensitivity" _col(35) "Specificity"   _col(50) "Observed" _col(65)  "Observed"    _col(80) "Positive"
dis as text _col(2) "ratio" _col(25) "%" _col(40) "%" _col(50) "risk ratio" _col(65)  "Case ratio" _col(80) "predictive"
dis as text  _col(80) "value (%)"
dis as text _col(2) _dup(100)"_"
dis as text _col(2) "Intraobserver TEM=0.24"
foreach j in 1 5 10 15 {
        clear
        allmicro `j'  .85098587   .85098587      .9942287    .9942287  /*Intraobserver TEM=0.24*/
qui set obs 4
qui gen zcase=1 if _n<=2
qui replace zcase=0 if _n>2
qui gen period=1 if _n==1 | _n==3
qui replace period=0 if period==.
qui gen nobs=.
qui replace nobs=round(oa,1) if _n==1
qui replace nobs=round(ob,1) if _n==2
qui replace nobs=round(oc,1) if _n==3
qui replace nobs=round(od,1) if _n==4
qui cs zcase period [fw=nobs]
dis as text _col(2) `j' _col(15) as res %4.2f trueRR _col(25) "85.1" _col(40) "99.4" as res _col(50) %6.4f r(rr) _col(65) as res %6.4f (oa/ob) _col(80) as res %4.1f  ppv
}
dis as text _col(2) _dup(100)"-"
dis as text _col(2) "Interobserver TEM=0.42"
foreach j in 1 5 10 15 {
        clear
        allmicro `j'   .7756936   .7756936    .98779317       .98779317  /*Intraobserver TEM=0.42*/
```

```
qui set obs 4
qui gen zcase=1 if _n<=2
qui replace zcase=0 if _n>2
qui gen period=1 if _n==1 | _n==3
qui replace period=0 if period==.
qui gen nobs=.
qui replace nobs=round(oa,1) if _n==1
qui replace nobs=round(ob,1) if _n==2
qui replace nobs=round(oc,1) if _n==3
qui replace nobs=round(od,1) if _n==4
qui cs zcase period [fw=nobs]
dis as text _col(2) `j' _col(15) as res %4.2f trueRR _col(25) "77.6" _col(40) "98.8" as res _col(50) %6.4f r(rr)
_col(65) as res %6.4f (oa/ob) _col(80) as res %4.1f  ppv
}
dis as text _col(2) _dup(100)"_"
dis as text _col(2) "Interobserver TEM=0.71"
foreach j in 1 5 10 15 {
	clear
	allmicro `j'   0.9125 0.9125 0.9723 0.9723  /*Interobserver TEM=0.71*/
qui set obs 4
qui gen zcase=1 if _n<=2
qui replace zcase=0 if _n>2
qui gen period=1 if _n==1 | _n==3
qui replace period=0 if period==.
qui gen nobs=.
qui replace nobs=round(oa,1) if _n==1
qui replace nobs=round(ob,1) if _n==2
qui replace nobs=round(oc,1) if _n==3
qui replace nobs=round(od,1) if _n==4
qui cs zcase period [fw=nobs]
dis as text _col(2) `j' _col(15) as res %4.2f trueRR _col(25) "91.2" _col(40) "97.2" as res _col(50) %6.4f r(rr)
_col(65) as res %6.4f (oa/ob) _col(80) as res %4.1f  ppv
}
dis as text _col(2) _dup(100)"_"
dis as text _col(2) "* RR: Risk ratio under perfect sensitivity and specificity"
end
doallmicro

/*8.2 Severe microcephaly*/
capture program drop sevmicro
quietly program define sevmicro
scalar z2=(32-(32-(3*1.2)))/1.2
scalar m3sdrisk=(1-normal(abs(z2))) /*This is the risk of severe microcephaly*/
scalar zrisk=0.10
scalar hrr=`1'                              /*This is the increase in risk of microcephaly among ZIKV infected mothers*/
scalar sensE1=`2'
scalar sensE0=`3'
scalar specE1=`4'
scalar specE0=`5'
scalar pop=10000
```

```
/*Background period*/
scalar e0z1m1=(pop*zrisk)*(m3sdrisk*`1')                    /*Cell a: Exposure=0; Zika=1; micro=1*/
scalar e0z1m0=(pop*zrisk)-e0z1m1                            /*Cell c: Exposure=0; Zika=1; micro=0*/
scalar e0z0m1=(pop*(1-zrisk))*(m3sdrisk)                    /*Cell b: Exposure=0; Zika=0; micro=1*/
scalar e0z0m0=(pop*(1-zrisk))-e0z0m1                        /*Cell d: Exposure=0; Zika=0; micro=0*/
scalar B=round((e0z1m1+e0z0m1),1)
scalar D=round((e0z1m0+e0z0m0),1)
scalar susceptible2=pop*(1-zrisk)
/*Outbreak period*/
scalar e1z1m1=(susceptible2*zrisk*2)*(m3sdrisk*`1')
scalar e1z1m0=(susceptible2*zrisk*2)- e1z1m1
scalar e1z0m1=(pop-(susceptible2*zrisk*2))*(m3sdrisk)
scalar e1z0m0=(pop-(susceptible2*zrisk*2))-e1z0m1
scalar A=round((e1z1m1+e1z0m1),1)
scalar C=round((e1z1m0+e1z0m0),1)
/*Correcting for misclassification error*/
scalar oa=(A*sensE1) + (C*(1-specE1))
scalar ob=(B*sensE0) + (D*(1-specE0))
scalar oc=(C*specE1) + (A*(1-sensE1))
scalar od=(D*specE0) + (B*(1-sensE0))
scalar ppv=(1-(((C*(1-specE1)) + (D*(1-specE0)))/(oa+ob)))*100  /*This is the positive predictive value*/
scalar trueRR=(A/(A+C))/(B/(B+D))                           /*RR if there were no misclassification errors*/
end

capture program drop dosevmicro
qui program define dosevmicro
dis as text _col(2) "Table 4. Observed relative increase in prevalence of newborn severe microcephaly and positive predictive"
dis as text _col(2) "values (PPV) in an outbreak as compared to a non-outbreak period of Zika virus (ZIKV) infection, by"
dis as text _col(2) "by hypothetical relative increase in the prevalence of microcephaly in newborn of ZIKV-infected mothers,"
dis as text _col(2)         "and by level of intra and inter-observer technical error of measurement (TEM) of head circumference."
dis as text _col(2) _dup(100)"_"
dis as text _col(2) "True risk" _col(15) "RR*"  _col(20) "Sensitivity" _col(35) "Specificity"  _col(50) "Observed" _col(65)  "Observed"    _col(80) "Positive"
dis as text _col(2) "ratio"  _col(25) "%"  _col(40) "%" _col(50) "risk ratio" _col(65)  "Case ratio" _col(80) "predictive"
dis as text _col(80) "value (%)"
dis as text _col(2) _dup(100)"_"
dis as text _col(2) "Intraobserver TEM=0.24"
foreach j in 1 5 10 15 {
        clear
        sevmicro `j'   .81904762   .81904762     .99943423    .99943423  /*Intraobserver TEM=0.24*/
qui set obs 4
qui gen zcase=1 if _n<=2
qui replace zcase=0 if _n>2
qui gen period=1 if _n==1 | _n==3
qui replace period=0 if period==.
```

```
qui gen nobs=.
qui replace nobs=round(oa,1) if _n==1
qui replace nobs=round(ob,1) if _n==2
qui replace nobs=round(oc,1) if _n==3
qui replace nobs=round(od,1) if _n==4
qui cs zcase period [fw=nobs]
dis as text _col(2) `j' _col(15) as res %4.2f trueRR _col(25) "81.9" _col(40) "99.9" as res _col(50) %6.4f r(rr)
_col(65) as res %6.4f (oa/ob) _col(80) as res %4.1f  ppv
}
dis as text _col(2) _dup(100)"-"
dis as text _col(2) "Interobserver TEM=0.42"
foreach j in 1 5 10 15 {
        clear
        sevmicro `j'  .74945055  .74945055      .9986842    .9986842  /*Intraobserver TEM=0.42*/
qui set obs 4
qui gen zcase=1 if _n<=2
qui replace zcase=0 if _n>2
qui gen period=1 if _n==1 | _n==3
qui replace period=0 if period==.
qui gen nobs=.
qui replace nobs=round(oa,1) if _n==1
qui replace nobs=round(ob,1) if _n==2
qui replace nobs=round(oc,1) if _n==3
qui replace nobs=round(od,1) if _n==4
qui cs zcase period [fw=nobs]
dis as text _col(2) `j' _col(15) as res %4.2f trueRR _col(25) "74.9" _col(40) "99.9" as res _col(50) %6.4f r(rr)
_col(65) as res %6.4f (oa/ob) _col(80) as res %4.1f  ppv
}
dis as text _col(2) _dup(100)"-"
dis as text _col(2) "Interobserver TEM=0.71"
foreach j in 1 5 10 15 {
        clear
        sevmicro `j'  .65934066 .65934066  .99594346  .99594346        /*Intraobserver TEM=0.71*/
qui set obs 4
qui gen zcase=1 if _n<=2
qui replace zcase=0 if _n>2
qui gen period=1 if _n==1 | _n==3
qui replace period=0 if period==.
qui gen nobs=.
qui replace nobs=round(oa,1) if _n==1
qui replace nobs=round(ob,1) if _n==2
qui replace nobs=round(oc,1) if _n==3
qui replace nobs=round(od,1) if _n==4
qui cs zcase period [fw=nobs]
dis as text _col(2) `j' _col(15) as res %4.2f trueRR _col(25) "65.93" _col(40) "99.59" as res _col(50) %6.4f r(rr)
_col(65) as res %6.4f (oa/ob) _col(80) as res %4.1f  ppv
}
dis as text _col(2) _dup(100)"_"
dis as text _col(2) "* RR: Risk ratio under perfect sensitivity and specificity"
end
```

dosevmicro

/*==*/

/*9.0 Estimating the minimum hypothetical relative increase in risk of GBS among Zika virus infected individuals, that would result in a detectable outbreak of GBS during an outbreak of Zika virus infection, after accounting for non-differential misclassification error of GBS, due to imperfect sensitivity and specificity of the GBS surveillance case definition. For formulae, see Lash et al. [21]. */

```
clear
capture program drop gbsx
quietly program define gbsx
clear
qui set obs 1
qui gen hrr=`1'                /*Hypothetical relative increase in GBS risk in ZIKV infected individuals*/
qui gen sensE1=`2'
qui gen sensE0=`3'
qui gen specE1=`4'
qui gen specE0=`5'
qui gen pop=10000000           /*10 million is the median population size in Latin American countries*/
qui gen gbsrisk=2/100000
qui gen zrisk=0.10
/*Background period*/
qui gen e0z1m1=(pop*zrisk)*(gbsrisk*`1' )          /*Cell a: Exposure=0; Zika=1; micro=1*/
qui gen e0z1m0=(pop*zrisk)-e0z1m1                  /*Cell c: Exposure=0; Zika=1; micro=0*/
qui gen e0z0m1=(pop*(1-zrisk))*(gbsrisk)           /*Cell b: Exposure=0; Zika=0; micro=1*/
qui gen e0z0m0=(pop*(1-zrisk))-e0z0m1              /*Cell d: Exposure=0; Zika=0; micro=0*/
qui gen B=round((e0z1m1+e0z0m1),1)
qui gen D=round((e0z1m0+e0z0m0),1)
qui gen susceptible2=pop*(1-zrisk)
/*Outbreak period: risk of ZIKV doubles, from 10% to 20%*/
qui gen e1z1m1=(susceptible2*zrisk*2)*(gbsrisk*`1')          /*Cell a: Exposure=1; Zika=1; micro=1*/
qui gen e1z1m0=(susceptible2*zrisk*2)- e1z1m1                /*Cell c: Exposure=1; Zika=1; micro=0*/
qui gen e1z0m1=(pop-(susceptible2*zrisk*2))*(gbsrisk)        /*Cell b: Exposure=1; Zika=0; micro=1*/
qui gen e1z0m0=(pop-(susceptible2*zrisk*2))-e1z0m1           /*Cell d: Exposure=1; Zika=0; micro=0*/
qui gen A=round((e1z1m1+e1z0m1),1)
qui gen C=round((e1z1m0+e1z0m0),1)
/*Expected observed numbers, accounting for misclassification*/
qui gen oa=(A*sensE1) + (C*(1-specE1)*0.0004469)
qui gen ob=(B*sensE0) + (D*(1-specE0)*0.0004469)  /*0.0004469 is the incidence of non-GBS non-diabetic
peripheral neuropathy*/
qui gen oc=(C*specE1) + (A*(1-sensE1))
qui gen od=(D*specE0) + (B*(1-sensE0))
end

capture program drop dogbs81
qui program define dogbs81
tempname memhold
tempfile q8
postfile `memhold' double(hrr specE1 rr rrpvalue81) using `q8'
        forvalues j = 1(1)15 {
        gbsx `j' .8206821 .8206821 0.806 0.806
```

```
            local a81=round(oa,1)
            local b81=round(ob,1)
            local c81=round(oc,1)
            local d81=round(od,1)
            qui csi `a81' `b81' `c81' `d81'
            qui gen rr81=r(rr)
            qui gen rrpvalue81=r(p)
            post `memhold' (hrr) (specE1) (rr81) (rrpvalue81)
            }
postclose `memhold'
use `q8', clear
gen yl=0.0227
label var yl "Outbreak detection cut-point"
label var hrr "Hypotethical risk ratio"
label var rrpvalue81 "Specificity=81%"
end

capture program drop dogbs89
qui program define dogbs89
tempname memhold
tempfile q8
postfile `memhold' double(hrr specE1 rr rrpvalue89) using `q8'
            forvalues j = 1(1)15 {
            gbsx `j' .8206821 .8206821 0.89 0.89
            local a89=round(oa,1)
            local b89=round(ob,1)
            local c89=round(oc,1)
            local d89=round(od,1)
            qui csi `a89' `b89' `c89' `d89'
            qui gen rr89=r(rr)
            qui gen rrpvalue89=round((r(p)),0.01)
            post `memhold' (hrr) (specE1) (rr89) (rrpvalue89)
            }
postclose `memhold'
use `q8', clear
gen yl=0.0227
label var yl "Outbreak detection cut-point"
label var hrr "Hypotethical risk ratio"
label var rrpvalue89 "Specificity=89%"
end

capture program drop dogbs92
qui program define dogbs92
tempname memhold
tempfile q8
postfile `memhold' double(hrr specE1 rr rrpvalue92) using `q8'
            forvalues j = 1(1)15 {
            gbsx `j' .8206821 .8206821 0.92 0.92
            local a92=round(oa,1)
            local b92=round(ob,1)
```

```
		local c92=round(oc,1)
		local d92=round(od,1)
		qui csi `a92' `b92' `c92' `d92'
		qui gen rr92=r(rr)
		qui gen rrpvalue92=r(p)
		post `memhold' (hrr) (specE1) (rr92) (rrpvalue92)
		}
postclose `memhold'
use `q8', clear
gen yl=0.0227
label var yl "Outbreak detection cut-point"
label var hrr "Hypotethical risk ratio"
label var rrpvalue92 "Specificity=92%"
end

capture program drop dogbs180
qui program define dogbs180
tempname memhold
tempfile q8
postfile `memhold' double(hrr specE1 rr rrpvalue180) using `q8'
		forvalues j = 1(1)15 {
		gbsx `j' .8206821 .8206821 1 1
		local a180=round(oa,1)
		local b180=round(ob,1)
		local c180=round(oc,1)
		local d180=round(od,1)
		qui csi `a180' `b180' `c180' `d180'
		qui gen rr180=r(rr)
		qui gen rrpvalue180=r(p)
		post `memhold' (hrr) (specE1) (rr180) (rrpvalue180)
		}
postclose `memhold'
use `q8', clear
label var hrr "Hypotethical risk ratio"
label var rrpvalue180 "Specificity=180%"
end

capture program drop dogbs100
qui program define dogbs100
tempname memhold
tempfile q8
postfile `memhold' double(hrr specE1 rr rrpvalue100) using `q8'
		forvalues j = 1(1)15 {
		gbsx `j' 1 1 1 1
		local a100=round(oa,1)
		local b100=round(ob,1)
		local c100=round(oc,1)
		local d100=round(od,1)
		qui csi `a100' `b100' `c100' `d100'
		qui gen rr100=r(rr)
```

```
		qui gen rrpvalue100=r(p)
		post `memhold' (hrr) (specE1) (rr100) (rrpvalue100)
		}
postclose `memhold'
use `q8', clear
scalar z1=(0 - (0+(1.96*1))/1)
gen pcut=(1-normal(abs(z1)))*2
label var pcut "Outbreak detection cut-point"
label var hrr "Hypotethical risk ratio"
label var rrpvalue100 "Specificity=100%"
end

capture program drop minrr
qui program define minrr
dogbs81
qui save gbs81, replace
dogbs89
qui save gbs89, replace
dogbs92
qui save gbs92, replace
dogbs180
qui save gbs180, replace
dogbs100
qui save gbs100, replace
qui merge 1:1 hrr using gbs180
qui drop _merge
qui merge 1:1 hrr using gbs92
qui drop _merge
qui merge 1:1 hrr using gbs89
qui drop _merge
qui merge 1:1 hrr using gbs81
dis as text _col(2) "Table 5. Minimum hypothetical risk ratio to detect an outbreak of GBS and expected 
observed"
dis as text _col(2) "risk ratios if ZIKV risk doubles during an outbreak, by level of specificity of GBS case"
dis as text _col(2) "detection, assuming a case detection sensitivity of 82.1%"
dis as text _col(2) _dup(89)"_"
dis _col(2) as text "Specificity"  _col(20) "Hypothetical"   _col(40) "Observed"
dis _col(5) as text "%"                                        _col(20) "risk ratio"            _col(40) "risk ratio"
dis as text _col(2) _dup(89)"_"
foreach i in 100 180 92 89 81 {    /*100--> Sens/spec=100/100; 180--> Sens/spec=82.1/100; 92--
>Sens/spec=82.1/91.97...*/
		qui sum hrr if rrpvalue`i'<pcut
		scalar minhrr=r(min)
		qui sum rr if rrpvalue`i'<pcut
		scalar minorr=r(min)
		dis as res _col(5) `i'              _col(20) minhrr  _col(40) %4.2f minorr
}
dis as text _col(2) _dup(90)"_"
end
minrr
```

```
/*==========================================================================================*/
/*10. Estimating the expected observed increase in risk of GBS among Zika virus infected individuals, during an outbreak of Zika virus infection, and the positive predictive value of a diagnosis of GBS, accounting for non-differential misclassification error of GBS, due to imperfect sensitivity and specificity of the GBS surveillance case definition. See Lash et al. for formulae. [21].*/
capture program drop gbs0
quietly program define gbs0
qui set obs 1
scalar hrr=`1'                  /*Hypothetical relative increase in GBS risk in ZIKV infected individuals*/
scalar sensE1=`2'               /*Sensitivity for GBS diagnosis in exposed period*/
scalar sensE0=`3'               /*Sensitivity for GBS diagnosis in non-exposed period; same as sensE1*/
scalar specE1=`4'               /*Specificity for GBS diagnosis in exposed period*/
scalar specE0=`5'               /*Specificity for GBS diagnosis in non-exposed period; same as sensE1*/
scalar pop=10000000             /*10 million is the median population size in Latin American countries*/
scalar gbsrisk=2/100000         /*Baseline risk of GBS*/
scalar zrisk=0.10               /*Baseline (non-outbreak) risk of ZIKV infection*/
scalar pnincid=`6'              /*95% confidence limits for incidence of peripheral neuropathy*/

 /*Calculating the number of individuals in each cell of the 2x2 table of outbreak-by-GBS*/
/*Non-outbreak=non-exposed period*/
scalar cases0=(pop*(zrisk*1)*hrr*gbsrisk) + (pop*(1-(zrisk*1))*gbsrisk)  /*non-outbreak*/
scalar ncases0=pop-cases0
scalar susceptible2=pop-(pop*(zrisk*1))
/*Outbreak=exposed period; risk of ZIKV doubles: zrisk*2 */
scalar cases1=(susceptible2*(zrisk*2)*hrr*gbsrisk) + (susceptible2*(1-(zrisk*2))*gbsrisk)     /*Outbreak*/
scalar ncases1=susceptible2-cases1

gen a=round(cases1,0)  /*Exposed cases*/
gen b=round(cases0,0)  /*Non-exposed cases*/
gen c=round(ncases1,0) /*Exposed non-cases*/
gen d=round(ncases0,0) /*Non-exposed non-cases*/

local a=int(cases1)    /*locals are created to be able to use csi, an inmediate command*/
local b=int(cases0)                     /*int() is used to get round numbers, because csi only accepts these numbers*/
local c=int(ncases1)   /*using ceil() instad of int() lead to the same risk ratios*/
local d=int(ncases0)

/*Accounting for misclassification due to imperfect sensitivity and specificity of GBS diagnosis. These would be the numbers observed in real live, i.e. the expected observed numbers, accounting for misclassification*/

scalar oa=(a*sensE1) + (c*(1-specE1)*(pnincid))  /*0.0004469 is the incidence of peripheral neuropathy*/
scalar ob=(b*sensE0) + (d*(1-specE0)*(pnincid))
scalar oc=(c*specE1) + (a*(1-sensE1))
scalar od=(d*specE0) + (b*(1-sensE0))
local oa=round(oa,1)
local ob=round(ob,1)
local oc=round(oc,1)
local od=round(od,1)
```

```
qui csi `a' `b' `c' `d'
scalar truerr=r(rr)
scalar lbtruerr=r(lb_rr)
scalar ubtruerr=r(ub_rr)
qui csi `oa' `ob' `oc' `od'
scalar obsrr=r(rr)
scalar lbobsrr=r(lb_rr)
scalar ubobsrr=r(ub_rr)
scalar ppv=((`a'+`b')/(oa+ob))*100
end
/*Creating a dataset with the results from the analysis*/
capture program drop dogbs
qui program define dogbs
tempname memhold
tempfile q8
postfile `memhold' double(hrr specE1 a b c d oa ob oc od  truerr lbtruerr ubtruerr obsrr lbobsrr ubobsrr ppv
pnincid) using `q8'
        forvalues i = 1(1)60 {                          /*Values of hypothetical risk ratio*/
                foreach j in 1 0.917 0.889 0.806 {      /*Values of specificity for GBS diagnosis*/
                        foreach k in 0.0003313 0.0005642 {      /*95% confidence limits for incidence of
                                                                peripheral neuropathy*/
                                clear
                                gbs0 `i' 0.821 0.821 `j' `j' `k'
        post `memhold' (hrr) (specE1) (a) (b) (c) (d) (oa) (ob) (oc) (od)  (truerr) (lbtruerr) (ubtruerr) (obsrr)
(lbobsrr) (ubobsrr) (ppv) (pnincid)
                }
        }
}
postclose `memhold'
use `q8', clear
label var hrr "Hypothetical risk ratio of GBS in ZIKV infected"
label var specE1  "Specificity of the diagnosis of GBS"
label var a "True exposed cases"            /*Exposed=outbreak period; cases=GBS*/
label var b "True non-exposed cases"        /*Non-exposed=non-outbreak period; cases=GBS*/
label var c "True exposed non-cases"        /*Exposed=outbreak period; non-cases=without GBS*/
label var d "True non-exposed non-cases"    /*Non-exposed=nonoutbreak period; non-cases=without GBS*/
label var oa  "Observed exposed cases" /*Corrected for misclassification; Exp=outbreak period; cases=GBS*/
label var ob  "Observed non-exposed cases"   /*Corrected for miscl; Non-exp=non-outbreak; cases=GBS*/
label var oc  "Observed exposed non-cases"     /*Corrected for miscl; Exp=outbreak; non-cases=without GBS*/
label var od  "Observed non-exposed non-cases"   /*Corrected for miscl; Non-exp=nonoutbreak; non-cases =
                                                  without GBS*/
label var truerr  "True risk ratio for GBS"         /*Without correction for misclassification*/
label var lbtruerr "Lower bound of truerr"
label var ubtruerr "Upper bound of truerr"
label var obsrr  "Observed risk ratio for GBS"      /*Accounting for misclassification*/
label var lbobsrr  "Lower bound of obsrr"
label var ubobsrr  "Upper bound of truerr"
label var ppv  "Positive predictive value"
replace ppv=100 if specE1==1
gen sensE1=0.821
```

```
label var sensE1 "Sensitivity of the diagnosis of GBS"
label var pnincid "Incidence of peripheral neuropathy"
gen fp=round((oa+ob)-(a+b),1)
replace fp=0 if fp<0
label var fp "Number of GBS false positive cases"
/*results for Table 6:  True and expected observed risk ratio of Guillain-Barré syndrome (GBS)
        during an outbreak of Zika virus (ZIKV) infection, by hypothetical increase in GBS risk among
        ZIKV infected individuals and by specificity and sensitivity of the GBS case definition used
        for surveillance.*/
list hrr specE1 pninci truerr obsrr ppv  fp if hrr==1   & pnincid==0.0003313, clean
list hrr specE1 pninci truerr obsrr ppv  fp if hrr==5   & pnincid==0.0003313, clean
list hrr specE1 pninci truerr obsrr ppv  fp if hrr==10 & pnincid==0.0003313, clean
list hrr specE1 pninci truerr obsrr ppv  fp if hrr==1   & pnincid==0.0005642, clean
list hrr specE1 pninci truerr obsrr ppv  fp if hrr==5   & pnincid==0.0005642, clean
list hrr specE1 pninci truerr obsrr ppv  fp if hrr==10 & pnincid==0.0005642, clean
end
dogbs

/*===================================================================================*/
/*11. Sample size needed to detect an outbreak of Zika virus infection if baseline risk is 10%, with 90%
power and 5% alpha error. The outbreak is defined as a risk equal or greater than the baseline risk plus two
standard deviations*/
dis as text _col(2) "The standard deviation of baseline risk is: " as res %4.2f (0.10*0.90)^0.5
dis as text _col(2) "The cut point to declare an outbreak is: " as res %4.2f 0.10+(2*((0.10*0.90)^0.5))
dis as text _col(2) "The minimum sample size for 90% power and 95% alpha error is: "
sampsi 0.10 0.16, power(0.9) onesample

/*===================================================================================*/
/*12. Estimating:
        a) The prevalence ratio of all microcephaly in newborn with low birth weight (<2,500 g) as compared
to normal weight, using data from different countries.
        b) The prevalence of low birth weight in Latin America, using data from Blanc et al.;[22] and
        c) The fraction of cases of microcephaly that may be attributed to low birth weight in Latin America,
using results from (a) and (b) and Levin's formula for population attributable risk. */

/*12.1. Estimating the prevalence ratio of microcephaly  */
/*Cuba: Pons Calderin et al. [23].
capture program drop cuba
qui program define cuba
clear
qui set obs 120
qui gen hc=33.7+1.1 * invnorm(uniform())
qui gen lbw=0
qui save t1, replace
clear
qui set obs 85
qui gen hc=31.0+1.6 * invnorm(uniform())
qui gen lbw=1
append using t1
qui gen micro=0
```

```
qui replace micro=1 if hc<31.8
qui capture cs micro lbw
qui gen rr=r(rr)
qui ci proportion lbw
qui gen plbw=r(proportion)
end
capture program drop simcuba
qui program define simcuba
qui simulate rr plbw, reps(`1') seed(2631530) nodots: cuba
end
capture program drop discuba
qui program define discuba
dis _n
dis as text _col(2) "Cuba, 2007; Pons Calderin5599"
qui centile _sim_1
scalar rr=r(c_1)
qui centile _sim_1, centile(2.5)
scalar lbrr=r(c_1)
qui centile _sim_1, centile(97.5)
scalar ubrr=r(c_1)
dis as text _col(2) "RR: " as res %4.2f rr " (" %4.2f lbrr ", " %4.2f ubrr ")"
qui centile _sim_2
scalar plbw=r(c_1)
dis as text _col(2)"Percent with low birth weight: " as res %4.2f plbw*100 "%"
end

/*Bangladesh: Ahmed et al. [24].
capture program drop bangla
qui program define bangla
clear
qui set obs 24
qui gen hc=27.9+2.9  * invnorm(uniform())
qui gen lbw=1
qui save t1, replace
clear
qui set obs 58
qui gen hc=30.5+2.0 * invnorm(uniform())
qui gen lbw=1
qui save t2, replace
clear
qui set obs 333
qui gen hc=32.2 + 2.0 * invnorm(uniform())
qui gen lbw=1
qui save t3, replace
clear
qui set obs 684
qui gen hc=33.6 + 1.0 * invnorm(uniform())
qui gen lbw=0
qui save t4, replace
clear
```

```
qui set obs 379
qui gen hc=34.7 + 1.5 * invnorm(uniform())
qui gen lbw=0
qui save t5, replace
clear
qui set obs 87
qui gen hc=35.33 + 1.1 * invnorm(uniform())
qui gen lbw=0
qui save t6, replace
clear
qui set obs 14
qui gen hc=37.6 + 2.8  * invnorm(uniform())
qui gen lbw=0
qui save t7, replace
append using t1
append using t2
append using t3
append using t4
append using t5
append using t6
qui gen micro=0
qui replace micro=1 if hc<31.8
qui capture cs micro lbw
qui gen rr=r(rr)
qui ci proportion lbw
qui gen plbw=r(proportion)
end
capture program drop simbangla
qui program define simbangla
qui simulate rr plbw, reps(`1') seed(2631530) nodots: bangla
end
capture program drop disbangla
qui program define disbangla
dis _n
dis as text _col(2) "Bangladesh, 2007; Taksande5603"
qui centile _sim_1
scalar rr=r(c_1)
qui centile _sim_1, centile(2.5)
scalar lbrr=r(c_1)
qui centile _sim_1, centile(97.5)
scalar ubrr=r(c_1)
dis as text _col(2) "RR: " as res %4.2f rr " (" %4.2f lbrr ", " %4.2f ubrr ")"
qui centile _sim_2
scalar plbw=r(c_1)
dis as text _col(2)"Percent with low birth weight: " as res %4.2f plbw*100 "%"
end

/*Bangladesh: Dhar et al. [25].    */
capture program drop bangla2
qui program define bangla2
```

```
clear
qui set obs 48
qui gen hc=31.5+1.6 * invnorm(uniform())
qui gen lbw=1
qui save t1, replace
clear
qui set obs 135
qui gen hc=33.2+1.1 * invnorm(uniform())
qui gen lbw=0
qui save t2, replace
clear
qui set obs 133
qui gen hc=34.5+1.0 * invnorm(uniform())
qui gen lbw=0
qui save t3, replace
append using t1
append using t2
qui gen micro=0
qui replace micro=1 if hc<31.8
qui capture cs micro lbw
qui gen rr=r(rr)
qui ci proportion lbw
qui gen plbw=r(proportion)
end
capture program drop simbangla2
qui program define simbangla2
qui simulate rr plbw, reps(`1') seed(2631530) nodots: bangla2
end
capture program drop disban2
qui program define disban2
dis _n
dis as text _col(2) "Bangladesh, 2000; Dahr5604"
qui centile _sim_1
scalar rr=r(c_1)
qui centile _sim_1, centile(2.5)
scalar lbrr=r(c_1)
qui centile _sim_1, centile(97.5)
scalar ubrr=r(c_1)
dis as text _col(2) "RR: " as res %4.2f rr " (" %4.2f lbrr ", " %4.2f ubrr ")"
qui centile _sim_2
scalar plbw=r(c_1)
dis as text _col(2)"Percent with low birth weight: " as res %4.2f plbw*100 "%"
end

/*India: Das et al. [26].*/
capture program drop india2
qui program define india2
clear
qui set obs 244
qui gen hc=28.44+3.46 * invnorm(uniform())
```

```
qui gen lbw=1
qui save t1, replace
clear
qui set obs 256
qui gen hc=34.33+2.16 * invnorm(uniform())
qui gen lbw=0
qui save t2, replace
append using t1
qui gen micro=0
qui replace micro=1 if hc<31.8
qui capture cs micro lbw
qui gen rr=r(rr)
qui ci proportion lbw
qui gen plbw=r(proportion)
end
capture program drop simindia2
qui program define simindia2
qui simulate rr plbw, reps(`1') seed(2631530) nodots: india2
end
capture program drop disindia2
qui program define disindia2
dis _n
dis as text _col(2) "India2, 2007; Das5588"
qui centile _sim_1
scalar rr=r(c_1)
qui centile _sim_1, centile(2.5)
scalar lbrr=r(c_1)
qui centile _sim_1, centile(97.5)
scalar ubrr=r(c_1)
dis as text _col(2) "RR: " as res %4.2f rr " (" %4.2f lbrr ", " %4.2f ubrr ")"
qui centile _sim_2
scalar plbw=r(c_1)
dis as text _col(2)"Percent with low birth weight: " as res %4.2f plbw*100 "%"
end

/*Uganda: Nabiwemba et al. [27]. */
capture program drop uganda
qui program define uganda
clear
qui set obs 85
qui gen hc=32.0+1.52 * invnorm(uniform())
qui gen lbw=1
qui save t1, replace
clear
qui set obs 621
qui gen hc=34.7+1.84 * invnorm(uniform())
qui gen lbw=0
append using t1
qui gen micro=0
qui replace micro=1 if hc<31.8
```

```
qui capture cs micro lbw
qui gen rr=r(rr)
qui ci proportion lbw
qui gen plbw=r(proportion)
end
capture program drop simuganda
qui program define simuganda
qui simulate rr plbw, reps(`1') seed(2631530) nodots: uganda
end
capture program drop disuganda
qui program define disuganda
dis _n
dis as text _col(2) "Uganda, 2007; Nabiwemba5606"
qui centile _sim_1
scalar rr=r(c_1)
qui centile _sim_1, centile(2.5)
scalar lbrr=r(c_1)
qui centile _sim_1, centile(97.5)
scalar ubrr=r(c_1)
dis as text _col(2) "RR: " as res %4.2f rr " (" %4.2f lbrr ", " %4.2f ubrr ")"
qui centile _sim_2
scalar plbw=r(c_1)
dis as text _col(2)"Percent with low birth weight: " as res %4.2f plbw*100 "%"
end

/*United States: Strauss et al. [28]. */
capture program drop ncppus
qui program define ncppus
clear
qui set obs 2719
qui gen hc=31.7+1.3 * invnorm(uniform())
qui gen lbw=1
qui save t1, replace
clear
qui set obs 43104
qui gen hc=34.0+1.3 * invnorm(uniform())
qui gen lbw=0
append using t1
qui gen micro=0
qui replace micro=1 if hc<31.8
qui capture cs micro lbw
qui gen rr=r(rr)
qui ci proportion lbw
qui gen plbw=r(proportion)
end
capture program drop simncppus
qui program define simncppus
qui simulate rr plbw, reps(`1') seed(2631530) nodots: ncppus
end
capture program drop disncppus
```

```
qui program define disncppus
dis _n
dis as text _col(2) "ncppus, 2007; Nabiwemba5606"
qui centile _sim_1
scalar rr=r(c_1)
qui centile _sim_1, centile(2.5)
scalar lbrr=r(c_1)
qui centile _sim_1, centile(97.5)
scalar ubrr=r(c_1)
dis as text _col(2) "RR: " as res %4.2f rr " (" %4.2f lbrr ", " %4.2f ubrr ")"
qui centile _sim_2
scalar plbw=r(c_1)
dis as text _col(2)"Percent with low birth weight: " as res %4.2f plbw*100 "%"
end

/*Getting the results for each country*/
capture program drop dothis
qui program define dothis
cuba
simcuba 1000
discuba
bangla
simbangla 1000
disbangla
bangla2
simbangla2 1000
disban2
india2
simindia2 1000
disindia2
uganda
simuganda 1000
disuganda
ncppus
simncppus 1000
disncppus
end
dothis

/*12.2 Taking a random effect average of the prevalence ratios estimated above*/
clear
input id str10 country year rr lbrr ubrr plbw
1 Cuba 2007 16.94 8.47 76.48 41.46
2 Bangladesh 2007 16.58 11.86 25.24 26.28
3 Bangla2 2000 10.79 6.65 19.54 15.19
4 India2  2007  6.93 5.12 10.29 48.80
5 Uganda 2007 7.85 5.19 11.80 12.04
6 US     1976 11.72 11.11 12.43 5.93
end
metan rr lbrr ubrr, random nograph
```

```
/*12.3 Estimating the prevalence of low birth weight in Latin America. Data come from Blanc et al. [22].*/
clear
input str30 country year pop rate
Bolivia         1994  3614          8.2
Bolivia         1998  6893          8.7
Brazil          1996  4782          10.4
Colombia        1990  3731          7.2
Colombia        1995  5050          8.7
Dominican       1991  3848          11.7
Dominican       1996  4379          12.5
Guatemala       1999  4545          13.0
Haiti           1994  3624          29.4
Nicaragua       1998  7992          13.0
Peru            1992  8540          10.2
Peru            1996  15639         11.1
end
capture program drop pla
qui program define pla
qui sum pop
gen w=1/(pop/r(sum))
qui ci means rate [weight=w]
dis as text _col(2) "Prevalence of low birth weight (<2,500 g) in Latin America: "  as res _col(30) %4.2f r(mean) " (" %4.2f r(lb)  ", " %4.2f r(ub) ")"
/*Estimating the fraction of cases of all microcephaly attributable to low birth weight in Latin America*/
dis as text "Proportion of cases of microcephaly attributable to low birth weight"
dis as text _col(2) "Lower bound: " as res %4.2f ((0.125*(7.34-1))/(1+(0.125*(7.34-1))))*100
dis as text _col(2) "Upper bound: " as res %4.2f ((0.125*(12.43-1))/(1+(0.125*(12.43-1))))*100
end
pla

/*=====================================================================================*/
/*13. Estimating the prevalence of GBS in Latin America during 2015-early 2016; using surveillance data reported by dos Santos et al. [29] Table 1*/
capture program drop doprevgbs
qui program define doprevgbs
clear
dis as text _col(2) "GBS incidence/100,000"
qui cii proportion 15203934 155
dis as text _col(2) "Brazil: " as res _col(30) %4.2f r(proportion)*100000 " (" %4.2f r(lb)*100000 ", " %4.2f r(ub)*100000 ")"
qui cii proportion 49529208 320
dis as text _col(2) "Colombia: " as res _col(30) %4.2f r(proportion)*100000 " (" %4.2f r(lb)*100000 ", " %4.2f r(ub)*100000 ")"
qui cii proportion 10652135 45
dis as text _col(2) "Dom Rep: " as res _col(30) %4.2f r(proportion)*100000 " (" %4.2f r(lb)*100000 ", " %4.2f r(ub)*100000 ")"
qui cii proportion 6426002  184
dis as text _col(2) "Salvador: " as res _col(30) %4.2f r(proportion)*100000 " (" %4.2f r(lb)*100000 ", " %4.2f r(ub)*100000 ")"
```

```
qui cii proportion 8423917  71
dis as text _col(2) "Honduras: " as res _col(30) %4.2f r(proportion)*100000 " (" %4.2f r(lb)*100000 ", " %4.2f
r(ub)*100000 ")"
qui cii proportion 548456   15
dis as text _col(2) "Suriname: " as res _col(30) %4.2f r(proportion)*100000 " (" %4.2f r(lb)*100000 ", " %4.2f
r(ub)*100000 ")"
qui cii proportion 31292702 684
dis as text _col(2) "Venezuela: " as res _col(30) %4.2f r(proportion)*100000 " (" %4.2f r(lb)*100000 ", " %4.2f
r(ub)*100000 ")"
end
doprevgbs
/*Data below are copied from above results*/
clear
input str10 country rate lbrate ubrate
Brazil          1.02 0.87 1.19
Colombia        0.65 0.58 0.72
DomRep          0.42 0.31 0.57
Salvador        2.86 2.46 3.31
Honduras        0.84 0.66 1.06
Suriname        2.73 1.53 4.51
Venezuela       2.19 2.03 2.36
end
metan rate lbrate ubrate, random nograph
exit
```

References

1. Pan American Health O: **Guidelines for Zika virus disease and complications surveillance**. Washington, D.C.: PAHO; 2016.
2. Duffy MR, Chen TH, Hancock WT, Powers AM, Kool JL, Lanciotti RS, Pretrick M, Marfel M, Holzbauer S, Dubray C *et al*: **Zika virus outbreak on Yap Island, Federated States of Micronesia**. *N Engl J Med* 2009, **360**(24):2536-2543.
3. Pepe MS: **The statistical evaluation of medical tests for classification and prediction**. Oxford: Oxford University Press; 2004.
4. Broemeling LD: **Bayesian biostatistics and diagnostic medicine**: CRC Press; 2007.
5. de Onis M: **Reliability of anthropometric measurements in the WHO Multicentre Growth Reference Study**. *Acta PaediatricaSupplement* 2006, **95**(s450):38-46.
6. Bhushan V, Paneth N: **The reliability of neonatal head circumference measurement**. *J Clin Epidemiol* 1991, **44**(10):1027-1035.
7. Fokke C, van den Berg B, Drenthen J, Walgaard C, van Doorn PA, Jacobs BC: **Diagnosis of Guillain-Barré syndrome and validation of Brighton criteria**. *Brain* 2014, **137**:33-43.
8. Choe YJ, Cho H, Bae GR, Lee JK: **Guillain-Barré syndrome following receipt of influenza A (H1N1) 2009 monovalent vaccine in Korea with an emphasis on Brighton Collaboration case definition**. *Vaccine* 2011, **29**(11):2066-2070.
9. Islam MB, Islam Z, Farzana KS, Sarker SK, Endtz HP, Mohammad QD, Jacobs BC: **Guillain-Barré syndrome in Bangladesh: validation of Brighton criteria**. *J Peripher Nerv Syst* 2016, **21**(4):345-351.
10. Katusic SK, Beard CM, Wiederholt WC, Bergstralh EJ, Kurland LT: **Incidence, clinical features, and prognosis in Bell's palsy, Rochester, Minnesota, 1968-1982**. *Ann Neurol* 1986, **20**(5):622-627.
11. Yanagihara N: **Incidence of Bell's palsy**. *Ann Otol Rhinol Laryngol Suppl* 1988, **137**:3-4.

12. Brandenburg NA, Annegers JF: **Incidence and risk factors for Bell's palsy in Laredo, Texas: 1974-1982**. *Neuroepidemiology* 1993, **12**(6):313-325.
13. Radhakrishnan K, Litchy WJ, O'Fallon WM, Kurland LT: **Epidemiology of cervical radiculopathy. A population-based study from Rochester, Minnesota, 1976 through 1990**. *Brain* 1994, **117 (Pt 2)**:325-335.
14. Vessey MP, Villard-Mackintosh L, Yeates D: **Epidemiology of carpal tunnel syndrome in women of childbearing age. Findings in a large cohort study**. *Int J Epidemiol* 1990, **19**(3):655-659.
15. de Krom MC, Knipschild PG, Kester AD, Thijs CT, Boekkooi PF, Spaans F: **Carpal tunnel syndrome: prevalence in the general population**. *J Clin Epidemiol* 1992, **45**(4):373-376.
16. Stevens JC, Sun S, Beard CM, O'Fallon WM, Kurland LT: **Carpal tunnel syndrome in Rochester, Minnesota, 1961 to 1980**. *Neurology* 1988, **38**(1):134-138.
17. Murakami S, Mizobuchi M, Nakashiro Y, Doi T, Hato N, Yanagihara N: **Bell palsy and herpes simplex virus: identification of viral DNA in endoneurial fluid and muscle**. *Ann Intern Med* 1996, **124**(1 Pt 1):27-30.
18. Beghi E, Kurland LT, Mulder DW, Nicolosi A: **Brachial plexus neuropathy in the population of Rochester, Minnesota, 1970-1981**. *Ann Neurol* 1985, **18**(3):320-323.
19. Baldereschi M, Inzitari M, Di Carlo A, Farchi G, Scafato E, Inzitari D, Group IW: **Epidemiology of distal symmetrical neuropathies in the Italian elderly**. *Neurology* 2007, **68**(18):1460-1467.
20. Rudolph T, Farbu E: **Hospital-referred polyneuropathies--causes, prevalences, clinical- and neurophysiological findings**. *Eur J Neurol* 2007, **14**(6):603-608.
21. Lash TL, Fox MP, Fink AK: **Misclassification**. In: *Applying quantitative bias analysis to epidemiologic data.* edn. Dordrecht: Springer; 2009: 79-108.
22. Blanc AK, Wardlaw T: **Monitoring low birth weight: an evaluation of international estimates and an updated estimation procedure**. *Bull World Health Organ* 2005, **83**:178-185d.
23. Pons Calderín O, Rivera Peña AL, Díaz Alonso G, Bacallao Gallesty J, Jiménez García R: **Valoración antropométrica en el recién nacido bajo peso**. *Revista Cubana de Medicina General Integral* 2000, **16**:18-24.
24. Ahmed FU, Karim E, Bhuiyan SN: **Mid-arm circumference at birth as predictor of low birth weight and neonatal mortality**. *J Biosoc Sci* 2000, **32**(4):487-493.
25. Dhar B, Mowlah G, Nahar S, Islam N: **Birth-weight status of newborns and its relationship with other anthropometric parameters in a public maternity hospital in Dhaka, Bangladesh**. *Journal of Health, Population and Nutrition* 2002:36-41.
26. Das S, Ghosh M, Mitra S, Chatterjee R, Bhattacharyya S, De AK, Chakraborty S: **Developing Nomogram to Estimate Birth Weight from Head Circumference and Mid-Upper Arm Circumference**. *J Trop Pediatr* 2012, **58**(4):307-310.
27. Elizabeth NL, Christopher OG, Patrick K: **Determining an anthropometric surrogate measure for identifying low birth weight babies in Uganda: a hospital-based cross sectional study**. *BMC Pediatr* 2013, **13**(1):54.
28. Strauss RS, Dietz WH: **Growth and development of term children born with low birth weight: Effects of genetic and environmental factors**. *The Journal of Pediatrics* 1998, **133**(1):67-72.
29. dos Santos T, Rodriguez A, Almiron M, Sanhueza A, Ramon P, de Oliveira WK, Coelho GE, Badaró R, Cortez J, Ospina M *et al*: **Zika Virus and the Guillain-Barré Syndrome -Case Series from Seven Countries**. *N Engl J Med* 2016, **375**:1598-1601.
